# Baxdrostat Efficacy and Safety in Uncontrolled and Resistant Hypertension

**DOI:** 10.1056/NEJMoa2507109

**Published:** 2025-08-30

**Authors:** John M. Flack, Michel Azizi, Jenifer M. Brown, Jamie P. Dwyer, Jakub Fronczek, Erika S.W. Jones, Daniel S. Olsson, Shira Perl, Hirotaka Shibata, Ji-Guang Wang, Ulrica Wilderäng, Janet Wittes, Bryan Williams, for the BaxHTN Investigators

**Affiliations:** 1 Departments of Medicine and Population Science and Policy, Division of General Internal Medicine - Hypertension Section, Southern Illinois University, Springfield, IL, USA; 2 Université Paris Cité, INSERM CIC1418, Paris, France; 3 AP-HP, Hypertension Department, Hôpital Européen Georges Pompidou, Paris, France; 4 Department of Medicine, Division of Cardiovascular Medicine, Brigham and Women’s Hospital, Boston, MA, USA; 5 Division of Nephrology and Hypertension, University of Utah, Salt Lake City, UT, USA; 6 Late-Stage Development, Cardiovascular, Renal, and Metabolism (CVRM), BioPharmaceuticals R&D, AstraZeneca, Warsaw, Mazowieckie, Poland; 7 Department of Medicine, Division of Nephrology and Hypertension, Groote Schuur Hospital, University of Cape Town, Cape Town, South Africa; 8 Late-Stage Development, Cardiovascular, Renal, and Metabolism (CVRM), BioPharmaceuticals R&D, AstraZeneca, Mölndal, Sweden; 9 Late-Stage Development, Cardiovascular, Renal, and Metabolism (CVRM), BioPharmaceuticals R&D, AstraZeneca, Gaithersburg, MD, USA; 10 Department of Endocrinology, Metabolism, Rheumatology and Nephrology, Faculty of Medicine, Oita University, Oita, Japan; 11 The Shanghai Institute of Hypertension, Ruijin Hospital, Shanghai Jiao Tong University School of Medicine, Shanghai, China; 12 Wittes LLC, Washington DC, USA; 13 University College London (UCL) Institute of Cardiovascular Science and National Institute for Health Research, UCL Hospitals Biomedical Research Centre, London, UK

## Abstract

**Background:**

Aldosterone dysregulation plays an important pathogenic role in hard-to-control hypertension. We tested the efficacy and safety of baxdrostat, an aldosterone synthase inhibitor, in patients with uncontrolled or resistant hypertension.

**Methods:**

This multinational, randomized, double-blind, placebo-controlled phase 3 trial recruited participants with seated office systolic blood pressure (seated-SBP) ≥140 mmHg and <170 mmHg, despite stable treatment with 2 (uncontrolled hypertension) or ≥3 (resistant hypertension) antihypertensive medications, including a diuretic. After a two-week placebo run-in, participants with seated-SBP ≥135 mmHg were randomized (1:1:1) to baxdrostat 1mg, baxdrostat 2mg, or placebo once daily for 12 weeks. The primary endpoint was change in seated-SBP from baseline to week 12.

**Results:**

794 participants were randomized to and received baxdrostat 1mg (n=264), baxdrostat 2mg (n=266), or placebo (n=264) in addition to background therapy. At week 12, change from baseline in least-squares mean seated-SBP for baxdrostat 1mg was –14.5 mmHg (95% confidence interval [CI], –16.5 to –12.5); baxdrostat 2mg, –15.7 mmHg (–17.6 to –13.7); versus placebo, –5.8 mmHg (–7.9 to –3.8). Estimated treatment differences for baxdrostat 1mg and 2mg relative to placebo were –8.7 mmHg (95% CI, –11.5 to –5.8; P<0.0001) and – 9.8 mmHg (–12.6 to –7.0; P<0.0001), respectively. Potassium >6.0 mmol/l occurred in 6 participants (2.3%) with baxdrostat 1mg, 8 (3.0%) with baxdrostat 2mg, and 1 (0.4%) with placebo.

**Conclusions:**

Baxdrostat added to background therapy resulted in a reduction in seated-SBP at 12 weeks compared with placebo in patients with uncontrolled or resistant hypertension.

(Funded by AstraZeneca and other; BaxHTN clinicaltrials.gov number, NCT06034743.)

**I**nappropriately elevated aldosterone production relative to patient sodium status is a key driver of hard-to-control (uncontrolled and resistant) hypertension and hypertension-mediated organ damage.^1–6^ Mineralocorticoid receptor antagonists (MRAs) can block the mineralocorticoid receptor-mediated pathophysiological effects of aldosterone but are underutilized due to dose-dependent adverse effects.^7–9^ Moreover, MRAs induce dose-related counter-regulatory increases in renin and circulating aldosterone concentrations that may stimulate MR-independent effects of aldosterone.^10–12^ An alternative therapeutic approach is direct inhibition of aldosterone synthase, which catalyzes the final three steps in aldosterone biosynthesis.^11^ Baxdrostat is a highly selective, potent aldosterone synthase inhibitor with a plasma half-life of approximately 30 hours, allowing once daily administration.^13,14^ In the 12-week phase 2 BrigHTN trial in patients with resistant hypertension, baxdrostat reduced seated office systolic blood pressure (seated-SBP) compared with placebo.^15^ However, in a phase 2 trial (HALO) in patients with uncontrolled hypertension, baxdrostat failed to show a difference in change from baseline in seated-SBP at week 8 versus placebo.^16^ In a more recent small study in patients with primary aldosteronism, baxdrostat substantially reduced seated-SBP.^17^ Here, we report the results of a longer-term, phase 3 trial assessing the efficacy and safety of baxdrostat in a broader population of patients with uncontrolled or resistant hypertension.

## Methods

### Trial Design, Population, and Procedures

BaxHTN is a phase 3, multicenter, randomized, double-blind, placebo-controlled trial, described previously.^18^ Participants were enrolled at 214 clinical sites across multiple countries (see Supplementary Appendix). Funded by AstraZeneca, the trial executive committee and AstraZeneca designed BaxHTN. Patients with hypertension were involved in the trial design, and the trial was conducted in accordance with the principles of the Declaration of Helsinki, the International Conference for Harmonization Good Clinical Practice guidelines, and applicable laws and regulations. Institutional Review Boards/Independent Ethics Committees approved the protocol (available at nejm.org), and all participants provided written, informed consent before enrollment. The trial was registered on clinicaltrials.gov on 09-13-23; https://clinicaltrials.gov/study/NCT06034743. Data were collected and analyzed by AstraZeneca. Programming was separately performed by two analysts to ensure accuracy. All authors had access to the data and the analyses; BW vouches for the completeness and accuracy of the data and analyses. BW wrote the first draft of the manuscript. Medical writing support was provided and funded by AstraZeneca (see Acknowledgments). All authors reviewed and edited the manuscript and agreed with the decision to publish. The study’s executive committee had professional services agreements applicable to their role in the study’s design, conduct, data analysis, and confidential information, and which supported the executive committee’s rights to publish the results of the study and use confidential information in connection with the performance of the services. The Sponsor retained the right to review publications for up to 60 days to confirm accuracy, safeguard confidential information and manage information relevant to patents.

The trial enrolled men and women aged ≥18 years with either uncontrolled or resistant hypertension, defined by a mean seated-SBP ≥140 mmHg and <170 mmHg despite treatment with maximally tolerated doses of either 2 (uncontrolled hypertension) or ≥3 (resistant hypertension) antihypertensive medications of different classes, including a diuretic, for ≥4 weeks before screening. Full inclusion and exclusion criteria are in the Supplementary Appendix.

After a two-week single-blind run-in period on placebo in addition to their stable background medications, participants were randomized if seated-SBP was ≥135 mmHg and there was good adherence to background antihypertensive therapy, as assessed by directly observed therapy and ≥80% adherence by pill count (see Supplementary Appendix).

The study consisted of four sequential parts over a total duration of 52 weeks (Fig. S1). Participants were divided into two cohorts based on time of randomization. The first 450 randomized patients were included in Cohort 1 and participated in all four parts of the study. All participants randomized thereafter were included in Cohort 2 and only participated in the first two parts of the study. This ensured sufficient power for the primary end point (end of part 1) and reduced the number of participants unnecessarily exposed to a second period of placebo treatment during the randomized withdrawal phase of the study (part 3).

Part 1 was a 12-week double-blind, randomized, placebo-controlled period, forming the basis of the primary outcome reported here. Participants were randomly assigned 1:1:1 to receive baxdrostat 1 mg, baxdrostat 2 mg, or placebo once daily. Randomization was stratified by baseline hypertension status (uncontrolled hypertension, resistant hypertension) and baseline seated-SBP (<145, ≥145 mmHg).

Part 2 (weeks 12–24) was a 12-week open-label phase designed to collect safety data and served as a run-in to Part 3. Participants who had received baxdrostat 2 mg in part 1 continued treatment, while those who had received baxdrostat 1 mg or placebo were re-randomized to baxdrostat 2 mg or standard-of-care (in a 4:1 or 1:4 ratio, respectively). Incorporation of the standard-of-care arm provided a comparator for longer term safety monitoring for the phases of the study that did not include placebo.

Part 3 (weeks 24–32) was an 8-week double-blind randomized withdrawal phase. Participants on baxdrostat 2 mg in part 2 were re-randomized (2:1) to baxdrostat 2 mg or placebo.

Part 4 (weeks 32–52) is an ongoing 20-week open-label phase to collect additional safety data for baxdrostat 2 mg. At the time of primary data lock, the two randomized controlled parts of the study, parts 1 and 3, were complete and are reported here, and a few participants from Cohort 2 were ongoing in the first open-label phase of the study (part 2).

Participants continued their background antihypertensive therapy throughout the trial. During double-blind periods, background therapy had to remain unchanged unless seated-SBP or diastolic blood pressure (DBP) exceeded 170 or 105 mmHg, respectively, in which case rescue therapy could be added at investigator discretion. During the open-label phases, adjustments to background antihypertensive therapy were permitted at investigator discretion; however, use of MRAs or potassium-sparing diuretics was only permitted in the standard-of-care arm.

During part 1, participants were evaluated at monthly visits prior to ingestion of study medications on the date of the visit. Attended seated-BP (Microlife WatchBP Office 2G), vital signs, medication list, and adverse events (AEs) were recorded. Blood samples were taken for serum creatinine, sodium, baxdrostat drug levels, aldosterone, and plasma renin activity (PRA), all measured at a central laboratory, blinded to randomization; serum potassium was measured at both local and central laboratories. In total, 34 study sites elected to undertake 24-hour ambulatory BP measurements at baseline and week 12. Additional details are provided in the Supplementary Appendix.

### End Points

The primary efficacy end point was the change in seated-SBP, from baseline to week 12, assessed for each baxdrostat group versus placebo. The secondary end points were: change in seated-SBP from week 24 to week 32, following randomized withdrawal of baxdrostat, in participants treated with baxdrostat 2 mg open-label daily for 12 weeks during part 2 (weeks 12 to 24) and then randomized to baxdrostat 2 mg versus placebo for 8 weeks; change in seated-SBP from baseline to week 12, in the resistant hypertension subpopulation; change in seated-DBP from baseline to week 12; and achieving seated-SBP <130 mmHg at week 12. Exploratory end points included change in ambulatory 24-hour and night-time average SBP from baseline to week 12, serum aldosterone concentrations and PRA.

Safety end points included AEs, vital signs, laboratory tests, and adjudicated major adverse cardiovascular events. AEs of special interest (AESI) included hyperkalemia (serum potassium >5.0 mmol/l), hyponatremia (serum sodium <135 mmol/l), and hypotension requiring medical intervention. An independent data monitoring committee reviewed the study data regularly.

### Statistical Analyses

Modified intention-to-treat populations were used for the analyses. The full analysis and safety analysis populations comprised all randomized participants who received ≥1 dose of study treatment. Analysis of the change in seated-SBP for randomized withdrawal period (part 3) baseline (week 24) to week 32 comprised all participants who were randomized and received ≥1 dose of study treatment in that period.

The primary analysis used analysis of covariance (ANCOVA) with treatment and hypertension status (uncontrolled, resistant) as factors and baseline seated-SBP as a covariate. All BP measurements were included regardless of study treatment discontinuation or need for rescue therapy. A hierarchical multiple testing procedure was used to control the familywise Type I error rate at 0.05 (two-sided) between the primary and secondary end points. Further details of the analysis plan, procedures for handling missing data, multiplicity procedure, sensitivity analyses and subgroup analyses are provided in the Supplementary Appendix.

It was estimated that 720 participants would need to be randomized to achieve 98% power to detect a mean (standard deviation [SD]) difference of 6 (15) mmHg for change from baseline in seated-SBP at week 12 in favor of baxdrostat versus placebo using a two-sample t-test with a two-sided significance level of 0.025. See Supplementary Appendix for further details.

Continuous variables are expressed as mean (SD) and median (interquartile range), and categorical variables as frequency (%). Between-group differences are expressed as least-squares (LS) means and 95% confidence intervals (CIs). All statistical analyses were performed using SAS software (SAS Institute Inc., Cary, NC, USA).

## Results

### Participant Characteristics

From November 2023 to February 2025, a total of 2591 participants were screened and 1109 were included in the placebo run-in; 796 were randomized and 794 received treatment with baxdrostat 1 mg (n=264), baxdrostat 2 mg (n=266) or placebo (n=264) (Fig. S2). Screen failure was most commonly due to failure to meet BP criteria. Sixty-six participants (8.3%) discontinued treatment during the 12-week double-blind period (part 1). The full analysis population included 794 participants. Of these, 754 (95%) achieved adherence to treatment rates ≥80% by tablet count for part 1.

Clinical characteristics at baseline were similar across treatment arms (Table 1, Table S1). The trial population was broadly representative of patients with uncontrolled and resistant hypertension (Table S2). Mean BP at baseline was 149/87 mmHg across groups. Background antihypertensive medications were similar at baseline and week 12 (Table S1, Table S3). During part 1, rescue medication was used by 7 (3%), 3 (1%) and 3 (1%) participants in the placebo, baxdrostat 1 mg and baxdrostat 2 mg groups, respectively.

A total of 18 (7%) participants in the placebo group, 13 (5%) in the baxdrostat 1 mg group and 16 (6%) in the baxdrostat 2 mg group had missing data that needed to be imputed for the primary endpoint (Table S4).

### Primary End Point

**Figure 1:**
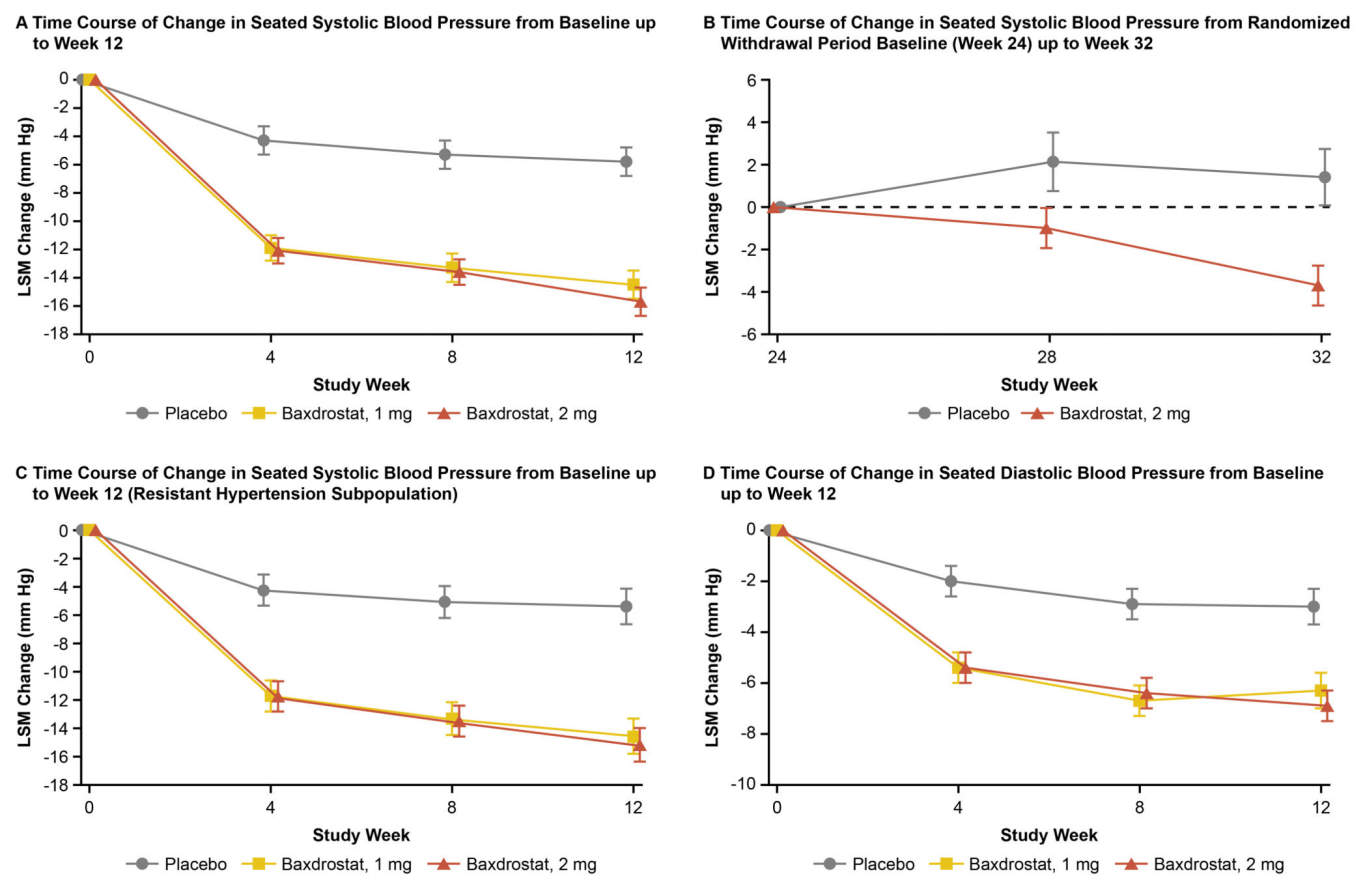
Blood Pressure Changes in Participants with Uncontrolled or Resistant Hypertension. The line graphs show time courses for LSM change from baseline in seated systolic blood pressure up to week 12 (Panel A), change from randomized withdrawal period baseline (week 24) in seated systolic blood pressure up to week 32 (Panel B), change from baseline in seated systolic blood pressure up to week 12 in the resistant hypertension subpopulation (Panel C), and change from baseline in seated diastolic blood pressure up to week 12 (Panel D). I bars on the line graphs indicate the standard error. LSM denotes least-squares mean.

**Figure 2:**
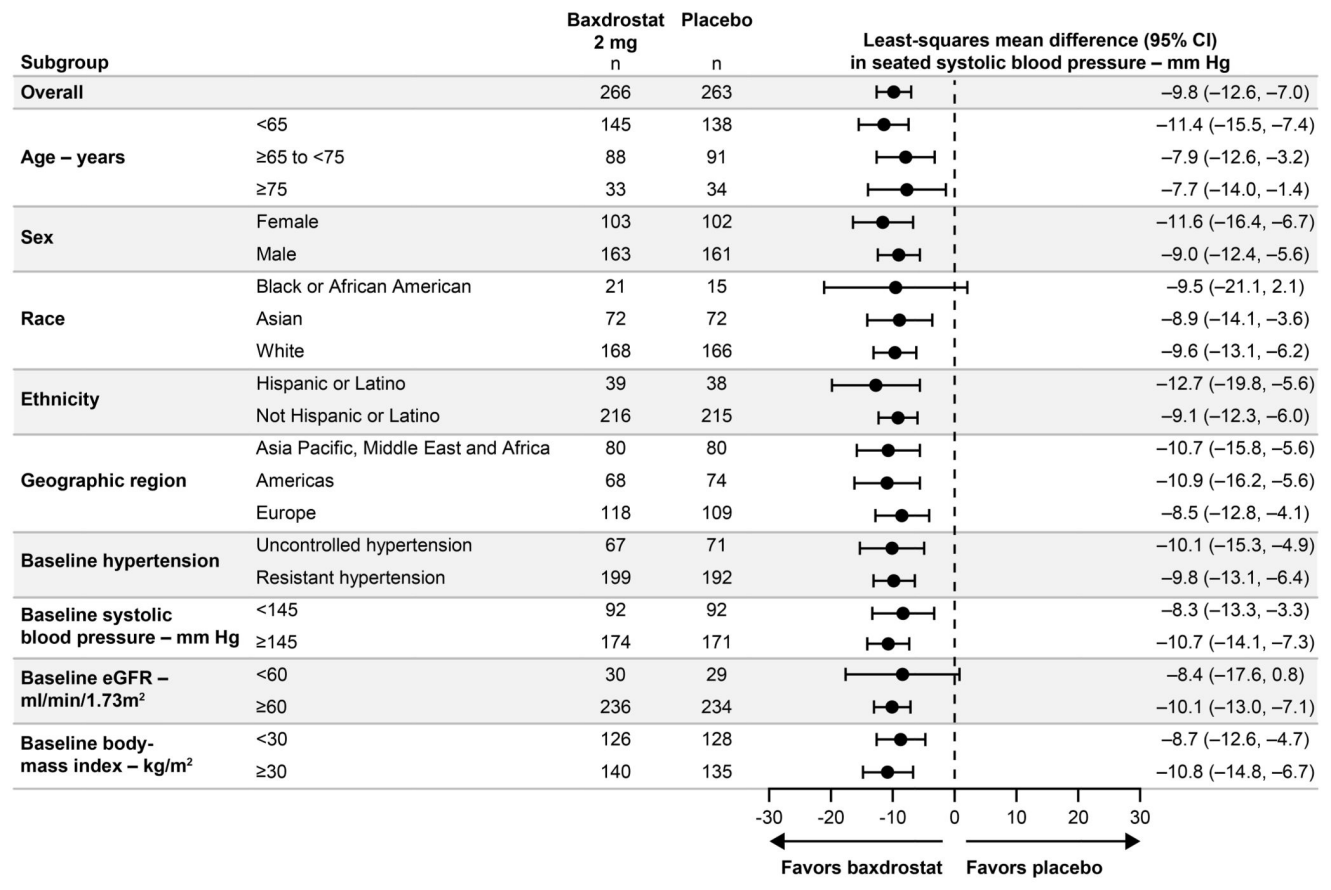
Change from Baseline in Seated Systolic Blood Pressure at Week 12 by Subgroup for Baxdrostat 2 mg versus Placebo. Forest plot shows least-squares mean difference versus placebo for change from baseline in seated systolic blood pressure at week 12 with baxdrostat 2 mg by subgroup. The analysis was performed within each subgroup category using an ANCOVA model with treatment and baseline hypertension (uncontrolled hypertension, resistant hypertension) as factors, and baseline seated systolic blood pressure value as a covariate. For the baseline hypertension subgroup analysis, the baseline hypertension factor was removed from the ANCOVA models for uncontrolled and resistant hypertension categories. Missing data were handled via multiple imputation. Circle denotes the point estimate. The widths of confidence intervals have not been adjusted for multiplicity and cannot be used to infer treatment effects. ANCOVA denotes analysis of covariance, CI confidence interval, and eGFR estimated glomerular filtration rate.

At week 12 (end of part 1), treatment with baxdrostat 1 mg and 2 mg resulted in a change from baseline in LS mean seated-SBP of –14.5 mmHg (95% CI, –16.5 to –12.5) and –15.7 mmHg (95% CI, –17.6 to –13.7), respectively, compared with a –5.8 mmHg (95% CI, –7.9 to –3.8) change with placebo (Table 2, Table S5 and Fig. 1). Estimated treatment differences of baxdrostat 1 mg and 2 mg relative to placebo were –8.7 mmHg (95% CI, –11.5 to –5.8; P<0.0001) and –9.8 mmHg (95% CI, –12.6 to –7.0; P<0.0001), respectively. Sensitivity analyses produced similar results (Table S6). Treatment effects by pre-specified subgroups for change in seated-SBP from baseline to week 12 are presented in Figure 2 and Figure S3.

### Secondary End Points

At the start of the 8-week randomized withdrawal period (part 3, week 24 to week 32), mean seated-SBP in those randomized to baxdrostat 2 mg and placebo groups was 133 mmHg. The change in LS mean seated-SBP during the randomized withdrawal period was –3.7 mmHg (95% CI, –5.5 to –1.9) with baxdrostat 2 mg and +1.4 mmHg (95% CI, –1.2 to 4.0) with placebo (estimated difference of –5.1 mmHg [95% CI, –8.3 to –1.9; P=0.0016]) (Table 2, Table S5 and Fig. 1).

In the resistant hypertension subpopulation, LS mean estimated placebo-corrected treatment differences in seated-SBP change at week 12 were –9.1 mmHg (95% CI, –12.6 to –5.7; P<0.0001) with baxdrostat 1 mg and –9.8 mmHg (95% CI, –13.1 to –6.4; P<0.0001) with baxdrostat 2 mg (Table 2, Table S5 and Fig. 1). For seated-DBP, LS mean estimated placebo-corrected treatment differences at week 12 were –3.3 mmHg (95% CI, –5.2 to –1.4; P=0.0008) with baxdrostat 1 mg and –3.9 mmHg (95% CI, –5.7 to –2.0; P<0.0001) with baxdrostat 2 mg (Table 2, Table S5 and Fig. 1).

The proportion of patients with controlled seated-SBP (<130 mmHg) at week 12 was 39.4% with baxdrostat 1 mg, 40.0% with baxdrostat 2 mg, versus 18.7% with placebo (odds ratios for BP control of 2.9 for both baxdrostat 1 mg and 2 mg versus placebo, both P<0.0001) (Table S5).

### Exploratory End Points

Data on exploratory end points, including ambulatory BP, serum aldosterone, plasma renin activity and pharmacokinetic analysis of baxdrostat drug levels, are provided in the Supplementary Appendix (Fig. S4–6; Table S7).

### Safety

During part 1, one death occurred in the placebo group. Serious AEs occurred in 5 (1.9%), 9 (3.4%) and 7 (2.7%) participants receiving baxdrostat 1 mg, baxdrostat 2 mg and placebo, respectively (Table 3; Table S8). AEs occurred in 125 participants (47.3%) receiving baxdrostat 1 mg, 119 (44.7%) receiving baxdrostat 2 mg, and 109 (41.3%) receiving placebo (Table 3). AEs were mostly mild, the most common being hyperkalemia, hyponatremia, hypotension, muscle spasms and dizziness (Table 3, Table S9). There were no reports of adrenal insufficiency.

Hyperkaemia (potassium levels >5.5mmol/l) occurred in 16/262 (6.1%), 29/261 (11.1%) and 1/260 (0.4) Potassium levels >5.0 mmol/l occurred in 61/256 (23.8%), 92/256 (35.9%) and 28/248 (11.3%) of participants receiving baxdrostat 1 mg, 2 mg and placebo, respectively (Table 3). Clinical intervention due to hyperkalemia (AESI) was reported in 7 (2.7%), 21 (7.9%) and 0 (0%) participants receiving baxdrostat 1 mg, 2 mg and placebo, respectively (Table 3). Serum potassium levels >6 mmol/l were recorded in a central laboratory for 6 (2.3%), 8 (3.0%) and 1 (0.4%) participants receiving baxdrostat 1 mg, baxdrostat 2 mg and placebo, respectively (Table 3, Table S10). Of these, 3 values (1.1%) for each of baxdrostat 1 mg and baxdrostat 2 mg were >6 mmol/l measured by a local laboratory on the same day (Table S11). Serum sodium concentrations <135 mmol/l occurred in 49 (19.1%) participants receiving baxdrostat 1 mg, 59 (22.8%) receiving baxdrostat 2 mg and 18 (7.0%) receiving placebo, up to week 12 (Table S10). However, few required medical intervention (2 [0.8%], 6 [2.3%] and 1 [0.4%] for baxdrostat 1 mg, 2 mg and placebo, respectively) (Table 3). Changes in serum potassium and sodium concentrations with baxdrostat occurred predominantly in the first two weeks and remained stable thereafter (Fig. S7 and S8). No changes were observed with placebo.

The mean change in estimated glomerular filtration rate (eGFR) from baseline to week 12 was –7.0 ml/min/1.73m^2^ (SD 12.8) and –6.9 (12.4) ml/min/1.73m^2^ in participants receiving baxdrostat 1 and 2 mg, respectively, and –0.1 (8.6) ml/min/1.73m^2^ in those receiving placebo (Fig. S9). The percent change in eGFR >30% that occurred on treatment was 12.6%, 15.6% and 1.5% and, ≥30% was 12.6%, 15.6% and 1.5% and ≥50% in 0.4, 1.5 and 1.1%, in participants receiving baxdrostat 1 and 2 mg, and placebo, respectively (Table S10). During the randomized withdrawal period (part 3), eGFR remained stable in the baxdrostat 2 mg group and returned towards baseline levels in the placebo group (Fig. S9). Two investigator-defined events of acute kidney injury were reported: one on placebo (0.4%) and one on baxdrostat 2 mg (0.4%).

## Discussion

In patients with uncontrolled or resistant hypertension, the addition of 1 mg or 2 mg daily doses of baxdrostat to background antihypertensive medication led to placebo-adjusted reductions in seated-SBP of 8.7 mmHg and 9.8 mmHg, respectively, after 12 weeks of treatment.

Our trial enrolled patients with hard-to-control BP despite receiving multiple antihypertensive medications. BP changes were similar in pre-specified subgroups, suggesting an important role for dysregulated aldosterone in the pathophysiology of both uncontrolled and resistant hypertension, and potentially a broader population of hypertensive patients.^5^ Though not subject to hypothesis testing, we noted reduced aldosterone and increased PRA levels that might suggest that baxdrostat may induce further urine sodium excretion in patients already treated with diuretics. We speculate that these changes may indicate that, despite renin-angiotensin-system inhibition, there is aldosterone breakthrough that may be contributing to the pathophysiology of hard-to-control BP.

Previous studies have shown that 5–10 mmHg reductions in SBP are associated with reduced risk of cardiovascular disease and death.^19,20^ The BP lowering effects of baxdrostat in our study were consistent with those reported for the aldosterone synthase inhibitor lorundrostat.^21,22^ In a phase 3 trial in patients with uncontrolled and resistant hypertension, the placebo-adjusted reduction in office SBP with lorundrostat 50 mg was –9.1 mmHg (95% CI, –13.3 to –4.9) at week 6 (primary endpoint).^21^

The current study provides additional data, including the impact of a randomized treatment withdrawal. At the start of the randomized withdrawal period (part 3), mean seated-SBP was 133 mmHg in those subsequently randomized to baxdrostat 2 mg and placebo. Over the next 8 weeks (weeks 24-to-32), the change in seated-SBP was –3.7 mmHg (95% CI, –5.5 to –1.9) in the baxdrostat group. Interestingly, in the placebo arm of the 8-week randomized withdrawal period, the change in seated-SBP was only +1.4 mmHg (95% CI, –1.2 to 4.0), despite the expected clearance of baxdrostat from the blood by 1 week.^14^ Moreover, serum aldosterone levels and PRA did not return fully to baseline. We speculate that the slow offset of baxdrostat’s effect on BP is consistent with its mechanism of action on sodium homeostasis.^13^ Other possible mechanisms include inhibition or reversal of aldosterone’s deleterious effects on the vasculature and sympathetic nervous system activity.^12,23^

In our study, the 12-week safety data with baxdrostat were generally consistent with lorundrostat clinical trials.^21,22^ The proportion of patients with serious AEs was low and similar across treatment arms. Hyperkalemia and hyponatremia occurred more frequently in the baxdrostat groups versus placebo, but there was a low incidence of hyperkalemia leading to discontinuation and a low rate of potassium measurements >6.0 mmol/l (Tables S10 and S11).

The mean change of –7.0 ml/min/1.73m^2^ in eGFR from baseline to week 12 observed in participants treated with baxdrostat occurred early, and during the randomized withdrawal period (part 3), eGFR returned towards baseline levels in the placebo group. These findings are consistent with functional eGFR changes due to the impact of BP lowering on renal perfusion.^24^

The present study has certain limitations. Ambulatory BP was measured in only a small number of participants. However, ambulatory BP is being measured in the ongoing 12-week Bax24 study (https://clinicaltrials.gov/study/NCT06168409),^18^ to be reported subsequently. There was a lower proportion of women and Black participants with hypertension enrolled than observed in the real world, although the proportion of Black participants was greater in North America (36%; Table S2). Otherwise, participant characteristics were largely aligned with those of patients with uncontrolled and resistant hypertension, and global recruitment was well balanced. Finally, medication adherence was not measured directly by objective methods throughout the study. However, the plasma levels of baxdrostat measured during the study indicate good adherence to study medication with ≤10% below the lower limit of quantification (Table S7).

In conclusion, baxdrostat when added to background antihypertensive therapy resulted in a reduction in seated-SBP at 12 weeks compared with placebo in a broad population of patients with uncontrolled and resistant hypertension.

Disclosure forms provided by the authors are available with the full text of this article at NEJM.org.

## Supplementary Material

Supplement

## Figures and Tables

**Table 1:** Demographics of Study Participants and their Clinical Characteristics at Baseline*

Characteristic	Placebo (N=264)	Baxdrostat, 1 mg (N=264)	Baxdrostat, 2 mg (N=266)
**Age** – yrs	61.9±11.6	59.8±11.8	61.8±11.7
**Male sex – no. (%)**	162 (61.4)	169 (64.0)	163 (61.3)
**Race or ethnic group – no. (%)^†^**			
White	167 (63.3)	165 (62.5)	168 (63.2)
Black	15 (5.7)	23 (8.7)	21 (7.9)
Asian	72 (27.3)	65 (24.6)	72 (27.1)
Other	8 (3.0)	10 (3.8)	2 (0.8)
Missing	2 (0.8)	1 (0.4)	3 (1.1)
Hispanic or Latino	38 (14.4)	27 (10.2)	39 (14.7)
**Seated blood pressure – mmHg**			
n	263	264	266
Systolic	149.0±8.7	149.7±10.1	149.1±9.1
Diastolic	85.8±10.5	88.0±10.5	85.8±10.5
**Body-mass index^‡^**	31.1±6.0	31.5±6.4	31.2±6.2
**Estimated glomerular filtration rate**– ml/min/1.73m^2§^	84.1±18.0	86.6±18.5	84.3±17.9
**Diabetes – no. (%)**	110 (41.7)	83 (31.4)	110 (41.4)
**Serum sodium – mmol/l**			
Mean±SD	139.6±2.5	139.9±2.6	139.8±2.5
Median (IQR)	140 (138,141)	140 (138,141)	140 (138,141)
**Serum potassium – mmol/l**			
Mean±SD	4.2±0.5	4.2±0.4	4.2±0.4
Median (IQR)	4.2 (3.9, 4.5)	4.2 (3.9, 4.4)	4.2 (3.9,4.5)
**Baseline hypertension^¶^ – no. (%)**			
Uncontrolled hypertension	71 (26.9)	77 (29.2)	67 (25.2)
Resistant hypertension	193 (73.1)	187 (70.8)	199 (74.8)

* Plus–minus values are mean ± standard deviation. Baseline characteristics are shown for the full analysis set (all randomized participants who received at least one dose of study intervention). Percentages may not total 100 because of rounding. To convert the values for sodium to milligrams, multiply by 23; to convert the values for potassium to milligrams, multiply by 39.

† Race and ethnic group were reported by the participant. The missing numbers included in the table relate to participants whose race information was not available.

‡ The body-mass index is the weight in kilograms divided by the square of the height in meters.

§ eGFR calculated according to CKD-EPI equation as per Inker LA et al.^25^

¶ Based on information collected in the medication case report form. CKD-EPI denotes Chronic Kidney Disease Epidemiology Collaboration, eGFR estimated glomerular filtration rate, IQR interquartile range, and SD standard deviation.

**Table 2:** Blood Pressure Changes with Baxdrostat – Primary Outcome and Secondary Outcomes (According to Hierarchical Order).

End Point	Placebo	Baxdrostat, 1 mg	Baxdrostat, 2 mg
Primary end point – change in seated-SBP from baseline to week 12*			
n	263	264	266
LS mean placebo-corrected difference (95% CI) – mmHg	–	–8.7 (–11.5 to –5.8)	–9.8 (–12.6 to –7.0)
P value	–	<0.0001	<0.0001
**Secondary end point – change in seated-SBP from randomized withdrawal period baseline (week 24) to week 32 (baxdrostat 2 mg versus placebo)†**			
n	85	NA	172
LS mean placebo-corrected difference (95% CI) – mmHg	–	NA	–5.1 (–8.3 to –1.9)
P value	–	NA	0.0016
**Secondary end point – change in seated-SBP from baseline to week 12 in the resistant hypertension subpopulation** ‡			
n	192	187	199
LS mean placebo-corrected difference (95% CI) – mmHg	–	–9.1 (–12.6 to –5.7)	–9.8 (–13.1 to –6.4)
P value	–	<0.0001	<0.0001
**Secondary end point – change in seated-DBP from baseline to week 12^§^**			
n	263	264	266
LS mean placebo-corrected difference (95% CI) – mmHg	–	–3.3 (–5.2 to –1.4)	–3.9 (–5.7 to –2.0)
P value	–	0.0008	<0.0001

* The analysis was performed on the full analysis set using an ANCOVA model with treatment and hypertension at baseline (uncontrolled hypertension, resistant hypertension) as factors, and baseline seated-SBP value as a covariate.

† The analysis was performed on the randomized withdrawal set as a pre-specified analysis for baxdrostat 2 mg versus placebo only, using an ANCOVA model with treatment and hypertension at baseline (uncontrolled hypertension, resistant hypertension) as factors, and randomized withdrawal period baseline seated-SBP value as a covariate.

‡ The analysis was performed on the full analysis set using an ANCOVA model with treatment as factor, and baseline seated-SBP value as a covariate. Only participants with resistant hypertension at baseline were included in the analysis.

§ The analysis was performed on the full analysis set using an ANCOVA model with treatment and hypertension at baseline (uncontrolled hypertension, resistant hypertension) as factors, and baseline seated-DBP value as a covariate. For all analyses except change in seated-SBP from randomized withdrawal period baseline (week 24) to week 32, missing data at week 12 following treatment discontinuation were imputed using a multiple imputation retrieved dropout method and missing data at week 12 following initiation of rescue medication were imputed using a multiple imputation washout method. For change in seated-SBP from randomized withdrawal period baseline (week 24) to week 32, a multiple imputation washout method was used at week 32 for missing data following both treatment discontinuation and rescue medication. Intercurrent events of deaths were handled using the hypothetical strategy (i.e., as if the subject had not died). P value is from the ANCOVA model for all analyses. ANCOVA denotes analysis of covariance, CI confidence interval, DBP diastolic blood pressure, LS least-squares, NA not applicable and SBP systolic blood pressure.

**Table 3:** Adverse Events During the 12-Week Double-Blind Treatment Period.

Event	Placebo (N=264)	Baxdrostat, 1 mg (N=264)	Baxdrostat, 2 mg (N=266)
		*Number (percent)*	
Any serious adverse event*	7 (2.7)	5 (1.9)	9 (3.4)
Death	1 (0.4)	0 (0.0)	0 (0.0)
Any adverse event	109 (41.3)	125 (47.3)	119 (44.7)
Moderate/severe	23 (8.7)	27 (10.2)	37 (13.9)
Severe	5 (1.9)	3 (1.1)	7 (2.6)
Any adverse event leading to discontinuation	5 (1.9)	7 (2.7)	12 (4.5)
Hyperkalemia leading to discontinuation	0 (0.0)	2 (0.8)	4 (1.5)
Adverse event of special interest (AESI) (i.e. resulted in clinical intervention)			
Hyperkalemia	0 (0.0)	7 (2.7)	21 (7.9)
Hyponatremia	1 (0.4)	2 (0.8)	6 (2.3)
Hypotension	2 (0.8)	5 (1.9)	6 (2.3)
Serum potassium – mmol/l^†^			
>5.0 mmol/l	28/248 (11.3)	61/256 (23.8)	92/256 (35.9)
>5.5 mmol/l	1/260 (0.4)	16/262 (6.1)	29/261 (11.1)
>6.0 mmol/l	1/262 (0.4)	6/262 (2.3)	8/263 (3.0)
>6.5 mmol/l	1/263 (0.4)	5/262 (1.9)	1/263 (0.4)

* One case of hyperkalaemia (baxdrostat 1 mg) and two cases of hyponatremia (baxdrostat 1 mg and 2 mg) were deemed by investigators to be possibly related to baxdrostat.

† Denominators are number of subjects per treatment group who at baseline did not already fulfil the specific row-criteria; for subjects with non-missing postbaseline value(s) but missing baseline value, the baseline value is assumed to not fulfil the specific row-criteria. Complete clinical chemistry treatment emergent abnormalities by predefined criteria up to week 12 are reported in Table S10.
