# Supplementary material for: Baxdrostat Efficacy and Safety in Uncontrolled and Resistant Hypertension
Source: N Engl J Med. Author manuscript; Available in PMC 2025 Oct 9. (PMC7618089; doi:10.1056/NEJMoa2507109)
Supplement: Supplement [file EMS207939-supplement-Supplement.pdf]

Supplementary Appendix

Flack JM, Azizi M, Brown JM, et al. Baxdrostat Efficacy and Safety in Uncontrolled and Resistant Hypertension.

This appendix has been provided by the authors to give readers additional information about the work.

Supplementary material – table of contents

	Page
BaxHTN Principal Investigators by Country (Centers)	3
Author Contributions	11
Supplementary Methods	12
Supplementary Results – Exploratory End Points	29
Figure S1. Study Design	31
Figure S2. Participant Disposition from Screening to Week 12 (A), and Throughout the Rest of the Study (B)	33
Figure S3. Change from Baseline in Seated Systolic Blood Pressure at Week 12 by Subgroup for Baxdrostat 1 mg versus Placebo	35
Figure S4. Change from Baseline to Week 12 in Ambulatory 24-hour Average SBP mm Hg (A) and Night-time Average SBP mm Hg (B)	36
Figure S5. Serum Aldosterone Levels (ng/dl) from Baseline to Week 12 (A), and from the Beginning (Week 24) to End (Week 32) of the Randomized Withdrawal Period (B)	38
Figure S6. Plasma Renin Activity (ng/ml/hr) from Baseline to Week 12 (A), and from the Beginning (Week 24) to End (Week 32) of the Randomized Withdrawal Period (B)	39

Figure S7. Serum Potassium Levels (mmol/l) from Baseline to Week 12	40
Figure S8. Serum Sodium Levels (mmol/l) from Baseline to Week 12	41
Figure S9. Estimated Glomerular Filtration Rate from Baseline to Week 12 (A), and from the Beginning (Week 24) to End (Week 32) of the Randomized Withdrawal Period (B)	42
Table S1. Full Demographics of Study Participants and their Clinical Characteristics at Baseline	43
Table S2. Representativeness of the Trial Population	47
Table S3. Background Antihypertensive Treatments at Week 12	51
Tale S4. Missing Data for the Primary End Point	53
Table S5. Blood Pressure Changes with Baxdrostat – Full Results for Primary Outcome and Secondary Outcomes (According to Hierarchical Order)	54
Table S6. Sensitivity Analyses of the Primary End Point (Full Analysis Set)	59
Table S7. Plasma Concentration of Baxdrostat Over Time (ng/ml)	61
Table S8. Serious Adverse Events by System Organ Class and Preferred Term During the 12-Week Double-Blind Treatment Period	63
Table S9. Adverse Events by System Organ Class and Preferred Term (Reported in >2% of Participants in Any Treatment Group) During the 12-Week Double-Blind Treatment Period	66
Table S10. Clinical Chemistry Treatment Emergent Abnormalities by Predefined Criteria During the 12-Week Double-Blind Treatment Period	68
Table S11. Assessment of Central Laboratory Potassium Measurements >6.0 mmol/l During the 12-Week Double-Blind Treatment Period	70
Supplementary References	71

BaxHTN Principal Investigators by Country (Centers)

Argentina
Alberto Liberman (Sanatorio Allende); Andrea Steinacher (Centro Medico Privado San Vicente Diabetes); Andres Francisco Alvarisqueta (Centro de Investigaciones Médicas); Celso García (Instituto de Investigaciones Clinicas San Nicolas); Diego Aizenberg (Centro Médico Viamonte); Fredy Ferre Pacora (Centro Medico Colon); Guillermo Mercau (Centro Modelo de Cardiologia); Gustavo Caruso (Hospital General de Agudos - José M. Ramos Mejía); Javier Zaidman (Centro de Investigación y Prevención Cardiovascular SA [CIPREC]); Juan Loureyro (INSTITUTO CAICI SRL.); Nicolas Federico Renna (Instituto de Cardiología Wolff; Batric Clínica Quirúrgica Bariátrica Omelanczuk); Sonia Sassone (Centro Medico Dra Laura Maffei-Investigación Clínica Aplicada)
Australia
Alexia Pape (Illawarra Shoalhaven Local Health District); Andrew Hamilton (Nightingale Research); Angela Makris (Liverpool Hospital); Bobby Chacko (John Hunter Hospital); Clara Chow (Westmead Hospital); Esther Davis (Victorian Heart Hospital); John Amerena (Barwon Health); Mark Arya (Australian Clinical Research Network); Mark Bloch (Holdsworth House Medical Practice); Markus Schlaich (Royal Perth Hospital); Michael Stowasser (Princess Alexandra Hospital); Muralikrishna Komala (Nepean Hospital); Om Narayan (The Cardiologists)
Austria
Johann Auer (Krankenhaus St. Josef Braunau GmbH); Martin Wiesholzer (Universitätsklinikum St. Pölten); Roza Badr-Eslam (Medizinische Universität Wien); Thomas Sturmberger (Ordensklinikum Linz GmbH)
Belgium
Hans T Kindt (AZ Maria Middelares Gent); Justine Huart (CHU de Liège); Peter Sinnaeve (UZ Leuven); Philippe Delmotte (CHU Helora); Sofie Brouwers (OLV Aalst); Tine De Backer (Universitair Ziekenhuis Gent [UZ Gent])
Bulgaria
Assen Goudev (University Multiprofile Hospital for Active Treatment Tsaritsa Yoanna – ISUL EAD); Blagovest Stoimenov (Diagnostic Consultation Center XX - Sofia EOOD); Dimitar Raev (MHAT Sveta Anna Sofia AD); Kiril Dzhomanov (University Multiprofile Hospital for Active Treatment Tsaritsa Yoanna – ISUL EAD); Snezhanka Tisheva-Gospodinova (Medical Center Kordis OOD); Stefan Naydenov (Diagnostic Consultation Center CONVEX EOOD)

Canada
Amritanshu Pandey (Cambridge Cardiac Care Centre); Andrew Yadegari (Heart Health Institute); Basel Bari (Markham HealthPlex Medical Centre); Daniel Gaudet (ECOGENE21 Clinical Trial Center CSSSC); Daniel Savard (CardioVasc HR); Gregory Hundemer (The Ottawa Hospital); James Cha (Dr James Cha); Jennifer Ringrose (Alberta Health Service); John Vyselaar (The Medical Arts Health Research Group); Luis Noronha (Diabetes Heart Research Centre); Shivinder Jolly (Clinical Research Solution Inc); Subodh Verma (Whitby Cardiovascular Institute); Swapnil Hiremath (The Ottawa Hospital); Yaariv Khaykin (Partners of Advanced Cardiac Evaluation [PACE])
Czech Republic
Jan Vaclavik (Fakultni nemocnice Ostrava); Jiri Krupicka (Medicus Services s.r.o.); Jiri Vesely (Edumed s.r.o.); Jitka Pruchova (Innera s.r.o.); Ladislav Busak (KardioBusak s.r.o.); Ondrej Jerabek (Kardiologicka ambulance s.r.o.); Vladimir Cech (Clinical Trials Service s.r.o.)
Denmark
Jesper Bech (Regionshospitalet Gødstrup); Michael Olsen (Holbæk Sygehus); Morten Lindhardt (Holbæk Sygehus); My Sofia Svensson (Aalborg University Hospital); Niels Henrik Buus (Aarhus University Hospital)
France
Antoine Cremer (CHU de Bordeaux); Béatrice Duly-Bouhanick (CHU de Toulouse); Benedicte Sautenet (CHRU de Tours / Hôpital Bretonneau); Christophe Mariat (Centre Hospitalier Universitaire de Saint-Étienne); Claire Bouleti (Centre Hospitalier Universitaire De Poitiers - Pôle Régional de Cancérologie); Gilles Montalescot (Hôpital Pitié-Salpêtrière); Jean Marc Boivin (Centre hospitalier régional et universitaire de Nancy); Jean-Michel Tartiere (Centre Hospitalier Intercommunal Toulon La Seyne-Sur-Mer Hôpital Saint Musse); Marilucy Lopez Sublet (Hôpital Avicenne); Michel Azizi (Hôpital Européen Georges Pompidou); Niki Procopi (Hôpital Pitié-Salpêtrière); Pascal Delsart (CHRU de Lille); Pierre-Jean Saulnier (Centre Hospitalier Universitaire De Poitiers - Pôle Régional de Cancérologie); Pierre-Yves Courand (Hôpital Lyon Sud)
Germany
Andrea Rinke (Siteworks GmbH); Andreas Hagenow (Zentrum für klinische Studien Südbrandenburg GmbH); Axel Schaefer (Medizentrum Essen Borbeck); Ayham Al-Zoebi (Kardiologische Praxis Wermsdorf); Bernhard Banas (Universitätsklinikum Regensburg); Bernhard Winkelmann (ClinPhenomics CVC GmbH); Christian Hugo (Universitätsklinikum Carl Gustav Carus der TU Dresden); Christoph Axthelm (Hausärztlich-Kardiologisches MVZ Am Felsenkeller); Dennis Kannenkeril (Universitaetsklinikum Erlangen); Felix Mahfoud (Universitätsklinikum des Saarlandes

Homburg/Saar); Florian Limbourg (Medizinische Hochschule Hannover, Hannover Medical School); Hannes Reuter (Universitätsklinikum Köln); Joachim Hoyer (Universitätsklinikum Gießen und Marburg GmbH); Joachim Weil (Sana Kliniken Lübeck); Johannes Stegbauer (Universitätsklinikum Düsseldorf); Klaus Busch (Diabeteszentrum Dortmund - Praxis Busch); Marcel Halbach (Universitätsklinikum Köln); Markus van der Giet (Charité - Universitätsmedizin Berlin); Martin Dürsch (ClinPhenomics CVC GmbH); Michael Böhm (Universitätsklinikum des Saarlandes Homburg/Saar); Michaela Hell (Universitätsmedizin der Johannes Gutenberg-Universität Mainz); Nicole Toursarkissian (Praxis Dr. med. Nicole Toursarkissian); Niels Menck (Helios Klinikum Erfurt); Niels-Christian Hoellger (Siteworks GmbH); Oliver Witzke (Universitätsklinikum Essen [AöR]); Philip Wenzel (Universitätsmedizin der Johannes Gutenberg-Universität Mainz); Ralf Dechend (Charité - Universitätsmedizin Berlin); Rüdiger Braun-Dullaeus (Universität Magdeburg); Sarah Rudolf (Universitätsklinikum Frankfurt); Sascha Brinkmann (Siteworks GmbH); Sebastian Cremer (Universitätsklinikum Frankfurt); Spasija Parizova (Herz- und Diabeteszentrum NRW); Stephan Schirmer (Kardiopraxis Schirmer); Thomas Forst (CRS Clinical Research Services Mannheim GmbH); Thomas Horacek (Evangelisches Krankenhaus Witten); Ulrike Rudolph (Universitätsklinikum Leipzig AöR); Wolfgang Jungmair (Kardiologische Praxis, Dr. med. Wolfgang Jungmair); Young Hee Lee-Barkey (Herz- und Diabeteszentrum NRW)
Hungary
Andras Vorobcsuk (Somogy Vármegyei Kaposi Mór Oktató Kórház, Da Vinci Magánklinika); Botond Literati-Nagy (DRC Gyógyszervizsgáló Központ); Gizella Pap (Stúdium Egészségház); Péter Légrády (Szegedi Tudományegyetem, Szent-Györgyi Albert Klinikai Központ); Tibor Kiss (Szabolcs-Szatmár-Bereg Vármegyei Oktatókórház); Zoltán Miklós Járai (Dél-budai Centrumkórház Szent Imre Egyetemi Oktatókórház); Zsolt Sarszegi (Coromed SMO Kft); Zsolt Zilahi (MediFarma-98 Kft.)
India
Aparna Kodre (Noble Hospital); Atul Abhyankar (Shri B.D. Mehta Mahavir Heart Institute); B K Srinivasa Sastry (Care Hospitals); Chakrabhavi Keshavamurthy (Manipal Hospitals); Dinesh Gautam (SMS Medical College, Jaipur); Govind Kulkarni (Sahyadri Speciality Hospital Deccan Gymkhana); Jitendra Pal Singh Sawhney (Sir Ganga Ram Hospital); Mullasari Sankardas (Madras Medical Mission); Phani Konide (Lalitha Super Specialty Hospital); Polavarapu Raghava Sarma (Lalitha Super Specialty Hospital); Puneet Saxena (SMS Medical College, Jaipur); Sandeep Bansal (Vardhman Mahavir Medical College & Safdarjung Hospital); Sankar Mandal (IPGME&R and Seth Sukhlal Karnani Memorial Hospital); Santhosh Satheesh (Jawaharlal Institute of Postgraduate Medical Education and Research [JIPMER]); Santosh Sinha (Ganesh Shankar Vidyarthi Memorial Medical College); SK Nasim (B. P. Poddar Hospital and Research Centre); Sreenivasa Lakshminarayanappa (Lifecare Clinic and Research Centre); Sunil Karna (Bhanubhai and Madhuben Patel Cardiac Centre. Shree Krishna Hospital and Medical Research Cente); Sunil

Kumar (JSS Hospital); Swapan Halder (NRS Medical College & Hospital); Tom Devasia (Manipal Centre for Clinical Research); Vijay Chopra (Max Super Speciality Hospital); Vimal Mehta (GB Pant Hospital)
Israel
Adi Leiba (Assuta Ashdod University Hospital); Benaya Rozen-Zvi (Rabin Medical Center); Gil Chernin (Kaplan Medical Center); Marina Tchircov (Rambam Health Care Campus); Nomy Levin-Iaina (Barzilai Medical Center); Yehonatan Sharabi (Sheba Medical Center)
Italy
Claudio Borghi (A.O.U. di Bologna – Policlinico Sant'Orsola-Malpighi); Franco Veglio (A.O.U. Città della Salute e della Scienza di Torino); Giuliano Tocci (Azienda Ospedaliero-Universitaria Sant'Andrea); Grzegorz Bilo (Istituto Auxologico Italiano); Leonardo Sechi (Azienda Sanitaria Universitaria Integrata di Udine); Maria Muiesan (ASST degli Spedali Civili di Brescia [Presidio Spedali Civil]); Marina Alimento (Monzino Cardiology Center [Centro Cardiologico Monzino]); Massimo Grimaldi (Ospedale Miulli); Stefano Carugo (Fondazione IRCCS Ospedale Maggiore Ca' Granda); Stefano Taddei (Azienda Ospedaliero Universitaria Pisana)
Japan
Atsushi Hirohata (The Sakakibara Heart Institute of Okayama); Hidenori Ishida (Akaicho Clinic); Hirokazu Yokoi (Rakuwakai Otowa Hospital); Hirotaka Nagashima (Tokyo Center Clinic); Katsuhiro Matsuda (Matsuda Internal and Cardiology Clinic); Kazuho Miyakoshi (Keigakukai Social Medical Corporation Minami Osaka Hospital); Kiyosue Arihiro (Tokyo Eki Center-Building Clinic); Kota Yamada (Tsuchiura Beryl Clinic); Naoki Itabashi (Itabashi Diabetes and Dermatology Medical Clinic); Tatsuya Nunohiro (Nagasaki Harbor Medical Center); Toru Arino (Yamaichi Bldg. Medical Clinic); Toshihiko Yasuda (Ishikawa Prefectural Central Hospital); Yuichi Yoshida (Oita University Hospital)
Malaysia
Chye Lee Gan (Hospital Melaka); Desmond Samuel (Hospital Miri); Frederick Walter De Rozario (Sarawak General Hospital); Meng Lee Chang (Hospital Seri Manjung); Mohd Faiz Faizul Fauzi (Hospital Raja Perempuan Zainab II); Muhamad Ali (Hospital Serdang); Zaid Azhari (Hospital Pakar Sultanah Fatimah)
Netherlands
Adriaan Kooy (Zuyderland Medisch Centrum Sittard-Geleen, Universitair Medisch Centrum Utrecht, Treant Zorggroep - Bethesda Ziekenhuis); Bert-Jan van den Born (Amsterdam Universitair Medisch

Centrum); Daan van Twist (Zuyderland Medisch Centrum Sittard-Geleen, Treant Zorggroep - Bethesda Ziekenhuis); Jaap Deinum (Radboud University Nijmegen Medical Centre); Sjaam Jainandunsings (Maasstad Ziekenhuis); Wilko Spiering (Amsterdam Universitair Medisch Centrum, Universitair Medisch Centrum Utrecht)
Poland
Aleksander Zurakowski (Małopolskie Centrum Sercowo – Naczyniowe PAKS); Andrzej Januszewicz (Instytut Kardiologii im. Prymasa Tysiąclecia Kard. St. Wysz); Andrzej Tykarski (Uniwersytecki Szpital Kliniczny w Poznaniu); Edyta Zbroch (Uniwersytecki Szpital Kliniczny w Białymstoku); Elżbieta Rosik (Centrum Medyczne PRATIA Gdynia); Jan Zbigniew Peruga (NZOZ Salus Med); Maciej Karcz (Gabinety Lekarskie 'profesorkarcz.pl'); Marek Rajzer (Samodzielny Publiczny Zakład Opieki Zdrowotnej Szpital Uniwersytecki w Krakowie); Michał Hoffmann (Uniwersyteckie Centrum Kliniczne); Paweł Grzelakowski (Centrum Kardiologii Paweł Grzelakowski); Piotr Sobieraj (MTZ Clinical Research Powered by PRATIA)
Slovakia
Daniela Vinanska (MEDISPOL, s.r.o.); Jan Fedacko (Cardio D&R s.r.o., Kardiologicka ambulancia); Lubomir Antalik (Nemocnica s poliklinikou Brezno, n.o.); Michal Banik (MEDI M&M s.r.o.); Peter Fulop (Interna SK, s.r.o.)
South Africa
Brian Rayner (University of Cape Town); Daniel Rudolf Malan (Prohealth and Wellness Clinic); Iftikhar Ebrahim (Raslouw Private Hospital); Lesley Burgess (Tread Research); Louis van Zyl (Clinical Projects Research); Lungisa Nojoko (Prohealth and Wellness Clinic); Maria Pretorius (Tygervalley Health Centre); Mark Abelson (Busamed Paardevlei Private Hospital); Mashudu Nethononda (CH Baragwanath Hospital); Naresh Ranjith (Dr Nash Ranjith Research Centre); Shaunagh Emanuel (Synopsis Research)
South Korea
Chan Joo Lee (Yonsei University Severance Hospital); Dae-Hee Kim (Asan Medical Center); Eung Ju Kim (Korea University Guro Hospital); Hae-Young Lee (Seoul National University Hospital); Jae-Hyeong Park (Chungnam National University Hospital); Jinho Shin (Hanyang University Seoul Hospital); Jung-Hyun Choi (Pusan National University Hospital); Sang Hyun Ihm (The Catholic University, Bucheon St Mary's Hospital); Seonghoon Choi (Hallym University Kangnam Sacred Heart Hospital); Soon Jun Hong (Korea University Anam Hospital); Wook Bum Pyun (Ewha Womans University Seoul Hospital); Wook-Jin Chung (Gachon University Gil Medical Center)

Spain
Alejandro de la Sierra (Hospital Mutua de Terrassa); Anna Oliveras (Hospital del Mar); Antonio Reyes Dominguez (Hospital Universitario Virgen de Valme); Esther Rubio (Hospital Universitario Puerta de Hierro Majadahonda); Fernando Jaén Águila (Hospital Universitario Virgen De Las Nieves); Francisco Martinez Deben (Complejo Hospitalario Universitario de Ferrol); José Antonio García Donaire (Hospital Clinico Universitario San Carlos); Jose Julian Segura de la Morena (Hospital Universitario 12 de Octubre); Juan Mediavilla García (Hospital Universitario Virgen De Las Nieves); Manuel Gorostidi Pérez (Hospital Universitario Central de Asturias); Miguel Camafort (Hospital Clínic Barcelona)
Sweden
Anna Stenborg (Akademiska Sjukhuset); Thomas Kahan (Danderyds sjukhus AB); Torbjörn Almgren (Sahlgrenska Universitetssjukhuset)
Taiwan
Chern-En Chiang (Veteran General Hospital Taipei); Chon-Seng Hong (Chi Mei Medical Center); Huang Chun-Yao (Taipei Medical University Hospital); Kuan-Cheng Chang (China Medical University Hospital); Kuo Feng-Yu (Kaohsiung Veterans General Hospital); Kwo-Chang Ueng (Chung Shan Medical University Hospital); Shih-Chung Huang (Kuang Tien General Hospital); Tsung-Hsien Lin (Kaohsiung Medical University Hospital); Yen-Hung Lin (National Taiwan University Hospital)
Thailand
Dilok Piyayotai (Thammasat University Hospital); Krit Leemasawat (Maharaj Nakorn Chiang Mai Hospital); Ply Chichareon (Songklanagarind Hospital, Prince of Songkla University); Poh Chanyavanich (Phrapokklao Hospital); Praew Kotruchin (Srinagarind Hospital, Khon Kaen University); Prin Vathesatogkit (Ramathibodi Hospital, Mahidol University); Sarawut Siwamogsatham (King Chulalongkorn Memorial Hospital, Chulalongkorn University); Weranuj Roubsanthisuk (Siriraj Hospital, Mahidol University)
Turkey
Abdulmecit Yildiz (Uludag University Medicine Faculty); Cagri Yayla (Ankara Bilkent City Hospital); Ergun Parmaksiz (Kartal Lutfi Kirdar Education and Research Hospital); Ersel Onrat (Afyonkarahisar Sağlık Bilimleri Üniversite Hastanesi); Ersin Simsek (T.C. Sağlık Bakanlığı İzmir Tepecik Eğitim ve Araştırma Hastanesi); Hamit Yildiz (Gaziantep University - Sahinbey Research and Training Hospital); Hilmi Erdem Sumbul (Adana City Training and Research Hospital); Mehmet

Özgeyik (Eskişehir Şehir Hastanesi); Özge Turgay Yıldırım (Eskişehir Şehir Hastanesi); Taner Sen (Kütahya Sağlık Bilimleri Üniversitesi); Ulver Derici (Gazi University Medical Faculty)
United Kingdom
Adrian Beltran-Martinez (Chesterfield Royal Hospital NHS Foundation Trust); Andrew Hill (Whiston Hospital); David Wingfield (Richford Gate Medical Practice); Gregory Lip (Whiston Hospital); Ivor Singh (West London NHS Trust); Jacob George (Ninewells Hospital & Medical School); Jaimini Tanna (Belmont Health Centre); James Case (GP – Concord Medical Centre); John Jackson (Pier Health Group); Linsday McCallum (NHS Greater Glasgow and Clyde); Manish Saxena (Barts Health NHS Trust); Nafeesa Arshad (GP – Concord Medical Centre); Pete Wilson (Middlewood Partnership); Samuel Davies (West Walk Surgery)
United States of America
Adam Karns (Entertainment Medical Group); Ahmed Awad (Clinical Research Consultants LLC); Alain Bouchard (Ascension Medical Group – St. Vincent); Alan Hinderliter (University of North Carolina at Chapel Hill); Alexander White (Progressive Medical Research); Andrew O'Shaughnessy (Nephrology Associates of Northern Indiana [NANI]); Anna Krawisz (Beth Israel Deaconess Medical Center); Brian Bostick (University of Missouri Health Care); Bryan Zweig (Henry Ford Health System); Carl Pepine (University of Florida); Charmaine Emelife (Southeast Kidney Associates); Christopher Romero (Headlands Research); Csaba Kovesdy (University of Tennessee Health Science Center); Daniel Johnson (Advanced Research Center, Inc.); David Bernard (CHEAR Center LLC); David Ramstad (Centricity Research Suffolk Family Medicine); David Tofovic (University of Illinois at Chicago); Deepika Jain (New Jersey Kidney Care); Ekamol Tantisattamo (UCI Health); Fadi Hage (Birmingham VA Medical Center); Harish Thakkar (Olympus Clinical Research); Heather Mascio (Clinical Renal Associates); Howard Rubenstein (The Center for Clinical Trials); Imran Chaudhary (University of Rochester Medical Center); Indu Poornima (Allegheny Health Network); Jalal Abbas (Clinical Research Institute of Arizona LLC); James Sample-Magee (East Coast Institute for Research - Jacksonville Southside); James Thrasher (Medical Investigations, Inc.); Jasjit Singh (The Nephrology Group Inc.); Jay Sandberg (Oakland Medical Research); Jeffrey Turner (Yale New Haven Health System); Jeffrey Wayne (Clinical Trials Research); Jeffrey Whittle (Zablocki VA Medical Center); John Delgado (Velocity Clinical Research); John Flack (Southern Illinois University School of Medicine - Hypertension Clinic); Joseph Miller (Henry Ford Health System); Justin Arambasick (The Research Group of Lexington LLC); Khaled Abdul-Nour (Henry Ford Health System); Kreton Mavromatis (Atlanta VA Medical Center); Larissa Kruger Gomes (Nephrology Associates, Inc.); Leonard Dunn (Clinical Research of West Florida Inc); Lyle Baker (Mayo Clinic); Manuel Montero (Eastern Nephrology Associates); Maria Gonzalez Suarez (Mayo Clinic); Marilyn King (Clearwater Cardiovascular Interventional Consultants); Matthew Tattersall (University of Wisconsin Hospital and Clinics); Minesh Rajpal (Southwest Kidney Institute); Musab Hommos (Mayo Clinic); Nabil Andrawis (Manassas Clinical

Research Ctr); Narender Goel (New Jersey Kidney Care); Nashwa Gabra (Burke Internal Medicine Inc); Nelson Kopyt (Northeast Clinical Research Center LLC); Ola Akinboboye (Laurelton Heart Specialists); Pablo Pergola (Clinical Advancement Center PLLC); Peter Santos (Arizona Kidney Disease & Hypertension Ctr); Rajat Barua (VA Medical Center – Kansas City); Randall Huling (Olive Branch Family Medical Center); Ravi Bhagwat (Cardiovascular Research of Northwest Indiana, L.L.C.); Richard Mohammed (Progressive Medical Research); Rocio Harbison (Juno Research LLC); Sadaf Elahi (Elixia at NANI); Samir Abdelshaheed (Centricity Research Suffolk Family Medicine); Scott Kuennen (Clay Platte Family Medicine); Sridhar Guduri (Centricity Research); Srinivas Hejeebu (University of Toledo Medical Center); Steven Barag (Rancho Cucamonga Clinical Research); Todd Gehr (Virginia Commonwealth University); Travis Taylor (South Texas Clinical Research); Vivek Bhalla (Stanford Health Care)
Vietnam
Anh Duong (Vinmec International Hospital); Binh Khong (Viet Duc Hospital); Dung Ho (Thong Nhat Hospital); Duy Le (BV Nguyen Tri Phuong – Hoi Dong Dao Duc); Hai Nguyen (Nhan Dan Gia Dinh Hospital); Huy Dinh (Tam Duc Hospital); Kieu Huynh (Tam Anh Hospital – Hochiminh); Nga Vu (Hanoi Heart Hospital – Trần Hưng Đạo); Trung Nguyen (Da Nang Hospital); Yen Nguyen (Tam Anh Hospital)

Author Contributions

All authors contributed to the conception or design of the work; and/or acquisition, analysis, or interpretation of data. BW produced the initial drafts supported by Jane Murphy and Mary Sayers of Ashfield MedComms, an Inizio Company. All authors contributed to drafting the manuscript or revising it critically for important intellectual content, and all gave final approval of the version to be published.

Supplementary Methods

Eligibility Criteria

Inclusion Criteria

1) Male or female participants ≥18 years old at the time of providing signed informed consent.
2) Mean seated office systolic blood pressure (seated-SBP) on attended automated office blood pressure monitor (Microlife WatchBP Office 2G) ≥140 mmHg and <170 mmHg at Screening.
3) Fulfilled at least one of the following two criteria:
    a) Participants in the uncontrolled hypertension subpopulation: a stable regimen of two antihypertensive medications from different therapeutic classes (at least one was a diuretic), at maximum tolerated doses in the judgment of the investigator, for at least 4 weeks before Screening. Beta-blockers used to treat other conditions (i.e., migraine, heart failure, coronary artery disease) were not counted as antihypertensive medications for the purpose of qualifying for the study.
    b) Participants in the resistant hypertension subpopulation: a stable regimen of ≥3 antihypertensive medications from different therapeutic classes (at least one was a diuretic) at maximum tolerated doses in the judgment of the Investigator, for at least 4 weeks before Screening. Beta-blockers used to treat other conditions (i.e., migraine, heart failure, coronary artery disease) were not counted as antihypertensive medications for the purpose of qualifying for the study.
4) Estimated glomerular filtration rate ≥45 ml/min/1.73m^2 at Screening, as calculated with the Chronic Kidney Disease Epidemiology Collaboration (CKD-EPI) 2021 equation.
5) Serum potassium level ≥3.5 and <5.0 mmol/l at Screening as determined by the central laboratory.

6) Morning cortisol levels >3 μg/dl as determined by the central laboratory.

7) Only female participants:

   Contraceptive used by females consistent with local regulations regarding the methods of contraception for those participating in clinical studies.

   i. Females not of child-bearing potential were defined as females who were either permanently sterilized (hysterectomy, bilateral oophorectomy, or bilateral salpingectomy), or who were postmenopausal. Females were considered postmenopausal if they have been amenorrheic for 12 months before the planned date of randomization without an alternative medical cause. The following age-specific requirements apply:

      a) Females <50 years old were considered postmenopausal if they have been amenorrheic for 12 months or more following cessation of exogenous hormonal treatment and follicle stimulating hormone levels in the postmenopausal range.

      b) Females ≥50 years old were considered postmenopausal if they have been amenorrheic for 12 months or more following cessation of all exogenous hormonal treatment.

   ii. Female participants of child-bearing potential using one highly effective form of birth control. A highly effective method of contraception was defined as one that can achieve a failure rate of less than 1% per year when used consistently and correctly. Females of child-bearing potential who were sexually active with a non-sterilized male partner agreed to use one highly effective method of birth control, as defined below, from 30 days before enrolment and throughout the study, and until at least 30 days after last dose of study intervention.

   iii. The following were not considered acceptable methods of contraception: periodic abstinence (calendar, sympto-thermal, post-ovulation methods), withdrawal (coitus interruptus), spermicides only, and lactational amenorrhea. Female condom and male condom were not to be used together.

iv. All females of child-bearing potential had a negative serum pregnancy test result at Screening and were not at stage of breastfeeding.

v. Highly effective birth control methods included: total sexual abstinence was an acceptable method provided it was the usual lifestyle of the participant (defined as refraining from heterosexual intercourse during the entire period of risk associated with the study treatments) ([periodic abstinence – e.g., calendar, ovulation, sympto-thermal, post-ovulation methods – declaration of abstinence for the duration of exposure to study intervention, and withdrawal were not acceptable methods of contraception]); a vasectomized partner; Implanon®; bilateral tubal occlusion; intrauterine device/levonorgestrel intrauterine system; Depo-Provera™ injections; oral contraceptive associated with inhibition of ovulation; and Evra Patch™, Xulane™, or NuvaRing®.

8) Capable of giving signed informed consent (including compliance with the requirements and restrictions listed in the Informed Consent Form and in the protocol).
9) Provision of signed and dated written Optional Genomics Initiative Research Information and Consent Form before the collection of samples for optional genomics initiative research that supports the Genomic Initiative. Participation was voluntary and if a participant declined to participate there was no penalty or loss of benefit.

Exclusion Criteria

1) As judged by the investigator, any evidence which in the investigator's opinion makes it undesirable for the participant to participate in the study.
2) Mean seated-SBP on automated blood pressure monitor ≥170 mmHg.
3) Mean seated diastolic blood pressure (seated-DBP) on automated blood pressure monitor ≥110 mmHg.

4) Current or prior treatment (within the 4 weeks before Screening) with an angiotensin-receptor blocker (ARB) and an angiotensin-converting enzyme inhibitor (ACEi), both taken simultaneously.
5) Serum sodium level <135 mmol/l at Screening, as determined by the central laboratory.
6) Had the following known secondary causes of hypertension: renal artery stenosis, uncontrolled or untreated hyperthyroidism, uncontrolled or untreated hypothyroidism, pheochromocytoma, Cushing's syndrome, aortic coarctation.
7) New York Heart Association functional heart failure class IV at Screening.
8) Medical history of stroke, acute coronary syndrome, hypertensive encephalopathy, or hospitalization for heart failure within 6 months before Screening.
9) Planned percutaneous coronary intervention/coronary artery bypass grafting, or percutaneous coronary intervention/coronary artery bypass grafting done within 6 months before Screening.
10) Known current severe left ventricular outflow obstruction, such as obstructive hypertrophic cardiomyopathy and/or severe aortic valvular disease.
11) Left bundle branch block and any cardiac arrhythmia requiring treatment.
12) Persistent atrial fibrillation.
13) Known severe hepatic impairment, defined as Child-Pugh Class C, based on records that confirm documented medical history.
14) Uncontrolled diabetes with HbA1c >10.0% (86 mmol/mol) at Screening.
15) Had a Screening QTcF value >470 msec.
16) Family history of Long QT syndrome.
17) Heart rate <45 or >110 beats/min in a resting position, as per vital signs assessment.
18) Participants with suspected severe cardiac hypertrophy.
19) Participants who were pregnant or breastfeeding.
20) Participants who had a diagnosis of adrenal insufficiency.
21) Any of the following related to COVID-19 infection:

i. Suspected (as judged by the principal investigator) or confirmed COVID-19 infection within the 4 weeks before Screening or at the Baseline Visit.
ii. Hospitalisation for COVID-19 within the last 12 weeks before Screening.

22) Prior medical treatment with any mineralocorticoid receptor antagonist, antiarrhythmic medications (beta-blockers and calcium channel blockers classified as Class II/IV antiarrhythmics used to treat hypertension, and digoxin were permitted), or potassium-sparing diuretics used within 4 weeks before Screening.
23) Treatment with potassium binders within 2 months before Screening.
24) Was expected to receive or was receiving any of the exclusionary drugs such as strong inducers of cytochrome P450 (CYP) 3A, chronic (taken more than three times a week for more than 3 months) use of nonsteroidal anti-inflammatory drugs (NSAIDs) (use of low-dose aspirin was permitted, as per medical judgment), mineralocorticoid receptor antagonists and/or chronic use of systemic steroids.
25) Drugs that prolong QT were avoided, if possible, and if there were other alternatives without the QT liability, these were preferred. If such QT-prolonging drugs were still needed, the investigator ensured appropriate monitoring of electrocardiograms and electrolytes were performed, as per clinical judgment.
26) Current or prior treatment within 6 months before Screening with cytotoxic therapy.
27) Treatment with potassium supplements were not prohibited but were continuously assessed and monitored throughout the trial.
28) Known hypersensitivity to baxdrostat or drugs of the same class or any of its excipients.
29) Participation in another clinical study with an investigational product administered in the 3 months before randomization into this study.
30) Participants working shifts (i.e., shifts that comprise working hours at different times on different days).

Randomization Criteria

These randomization criteria only applied to the initial randomization of the study. Participants were eligible to be randomized to a treatment group only if all the following criteria applied:

1) Mean seated-SBP on automated office blood pressure monitor of ≥135 mmHg at the Baseline Visit
2) Had 80–120% adherence to placebo during the run-in period, based on pill counts on the morning of randomization.
3) Had no change in background therapy regimen and dose consisted of either two antihypertensive medications (including a diuretic) for participants in the uncontrolled hypertension subpopulation, or ≥3 antihypertensive medications (including a diuretic) for participants in the resistant hypertension subpopulation, for at least 4 weeks before randomization. Beta-blockers used to treat other conditions (i.e., migraine, heart failure, coronary artery disease) were not counted as antihypertensive medications for the purpose of qualifying for this study.
4) Demonstrated good adherence to the prescribed antihypertensive medications by direct observed therapy during Visit 2 (see separate section on direct observed therapy).

Prohibited Concomitant Medications

The following medications were prohibited during the study, except in the standard-of-care group:

- Simultaneous use of ARB and ACEi.
- Potassium-sparing diuretics (e.g., amiloride, triamterene) and direct renin inhibitor (e.g., Aliskiren).
- Potassium binders (prohibited at Screening but can be started as a corrective action during the study).

- Tacrolimus, calcineurin inhibitors, and cyclosporin. Topical/inhaled immunosuppressants were permitted.
- Mineralocorticoid receptor antagonists or aldosterone antagonists (e.g., eplerenone, finerenone, spironolactone).
- Systemic corticosteroids at any dose (e.g., prednisone, prednisolone, dexamethasone [topical and inhaled steroids were allowed]).
- Treatment with potassium supplements were not prohibited but were continuously assessed and monitored throughout the trial.
- Chronic NSAID use. If occasional NSAID usage (less than three times a week for less than 3 months) was required, the investigator had to determine the extent and frequency of additional monitoring based on their clinical judgement, taking into account factors such as kidney function, hydration status, NSAID dose, concurrent medication usage, and severity of concomitant diseases.
- Strong CYP3A4 inducers (e.g., apalutamide, avasimibe, carbamazepine, enzalutamide, lumacaftor, mitotane, phenytoin, rifampin, rifapentine, St. John’s wort).

Randomization Methods and Blinding

Participants were assigned to randomized study intervention using an automated interactive response technology/randomization and trial supply management system (IRT/RTSM) at three randomization stages during the study (Fig. S1): 1) after the run-in period to allocate participants to baxdrostat 1 mg, baxdrostat 2 mg, or placebo; 2) after the initial 12-week double-blind period to allocate participants to baxdrostat 2 mg or standard-of-care; 3) after the 12-week open-label period of baxdrostat 2 mg to allocate participants to baxdrostat 2 mg or placebo.

Randomization was performed in balanced blocks of fixed size, with randomization codes being computer generated and loaded into the interactive web response system.

Randomization at the start of part 1 of the study had a block size of 6. Randomization at the start of part 2 had a block size of 1 for baxdrostat 2 mg and 5 for baxdrostat 1 mg and placebo. Randomization at the start of part 3 had a block size of 3. All randomizations were performed by authorized site personnel using the automated IRT/RTSM; use of this system ensured allocation concealment. To avoid over- or under-representation, the number of randomized participants in the uncontrolled hypertension and resistant hypertension populations was monitored to ensure a minimum of 40% participants and could be capped if the predetermined limit was reached.

The study treatment and placebo tablets and blister wallets were identical in physical appearance. During double-blind periods, neither the participants, sponsor, or investigators knew the intervention assignments until study completion. During the open-label periods, the participants, sponsor, and investigators were aware of intervention assignments.

The IRT/RTSM was programmed with blind-breaking instructions. The randomization code was not to be broken except in medical emergencies when the appropriate management of the participant required knowledge of the treatment randomization. The Investigator was obligated to document and report any blind breaks to the sponsor without revealing the treatment given to the participant to other clinical sites or blinded study personnel. If a participant's intervention assignment was unblinded, the sponsor was to be notified within 24 hours. The date and reason for the unmasking were recorded in the source documentation.

Drug Administration

Baxdrostat 1 mg, baxdrostat 2 mg, and placebo tablets were administered orally, one tablet per day, preferably in the morning. Study intervention was administered at site after completing blood pressure assessments at all visits (i.e., blood pressure was measured at trough). On all other days, the study intervention was self-administered by participants at

home. At visit 2 during the run-in period, participants took placebo and their existing background antihypertensive medications as part of direct observed therapy.

Direct Observed Therapy

Direct observed therapy was performed during the run-in visit as follows:

- On the run-in visit day (visit 2), participants did not take their background antihypertensive treatments at home.
- During the run-in visit, participants had their seated-SBP measured (i.e., at trough). Following this, study personnel administered the participant’s background antihypertensive treatments and witnessed the participant taking the medications along with placebo.
- The participant was then monitored for any adverse effects for at least 1 hour. Thereafter, the participant’s SBP was measured using ambulatory blood pressure measurement (ABPM), or at site, the general practitioner, pharmacy or home at 4 to 12 hours after medication intake.
- For SBP measured using ABPM, the first 12 SBP values were discarded. The following 15 SBP values (or less if recording ended earlier) were used to assess the patient’s response to direct observed therapy assessment. More than half of the SBP values should have been ≥130 mmHg. If at least half of the SBP values were <130 mmHg, the participant was considered a screen failure and discontinued from the study.
- If SBP was measured at site, the general practitioner, pharmacy or home, the SBP measurement should have been ≥130 mmHg. If the SBP value was <130 mmHg, the participant was considered a screen failure and discontinued from the study.

Assessment and Management of Hyperkalemia

Participants with potassium levels between 5.5 and 5.9 mmol/l remained on study intervention; their potassium level was rechecked within 72 hours by the local and central laboratory. If the potassium level was confirmed as ≥5.5 mmol/l, the study intervention was temporarily stopped. Participants with potassium levels ≥6.0 mmol/l temporarily stopped study intervention immediately, and levels were rechecked within 72 hours by the local and central laboratory. The study intervention could be restarted when the potassium level was ≤5.0 mmol/l. The study intervention was permanently discontinued if a participant experienced a recurrent hyperkalemia event (potassium ≥6.0 mmol/l) and there was no explanation for the recurrent event other than restarting study intervention.

For the 12-week double-blind treatment period (weeks 0–12), potassium measurements >6.0 mmol/l based on central laboratory measurements were further assessed as follows. If both central and local laboratory potassium values were >6.0 mmol/l on the same day, the central potassium value was classified 'confirmed'; if the local potassium value was ≤6.0 mmol/l, the central potassium value was classified 'not confirmed'; if the local potassium value was not available, the central potassium value was classified as 'undetermined'.

Monitoring of Adrenal Insufficiency

Morning cortisol was measured during screening and only patients with morning cortisol levels >3 µg/dl were eligible for inclusion. During the study, investigators monitored participants for symptoms associated with adrenal insufficiency. If such symptoms arose, an ad-hoc morning cortisol measurement and/or an ACTH stimulation test were performed. Centrally, clustering of adverse events associated with potential adrenal insufficiency were systemically monitored per participant to further ensure that any case of adrenal insufficiency was detected.

Blood Pressure Measurements

Seated Office Blood Pressure

Participants were evaluated during morning clinic visits at baseline and at prespecified intervals after randomization. Seated-BP measurements were obtained using an automated Microlife WatchBP Office 2G device after a participant had been seated for >5 minutes. All personnel that used the devices were trained and certified on the device used in the study. The system also includes several checks that detect poor quality of assessment, e.g., movement of the participant while measuring blood pressure. The measurement was electronically recorded and transferred to central database using centrally provided laptops with dedicated software provided by Clario BP Services without any possibility for adjustment by the site personnel or the sponsor.

Three BP measurements approximately 1 minute apart were obtained using the same arm; mean seated-BP was the average of the last two measurements at any visit. If multiple mean seated blood pressure readings were obtained during the baseline visit, the last mean seated-BP was used. For any post-baseline visit, the first mean seated-BP value was used if the difference between the last two measurements was within 20 mmHg. If the difference was >20 mmHg, the first mean seated-BP with the difference within 20 mmHg was used. If all available mean seated-BP values had a difference >20 mmHg between the last two measurements, the last available mean seated-BP was used.

Ambulatory Blood Pressure Monitoring

Participation in the ABPM assessment was optional but highly encouraged. For these assessments, participants wore the ABPM device (Microlife WatchBP O3) for a minimum of 25 hours (the first hour of blood pressure readings after device fitting was not included in the analyses). The ABPM device was fitted to study participants on the non-dominant arm. If a session failed to meet acceptability criteria it was not repeated.

ABPM assessments were conducted as follows:

- Run-in (visit 2): the ABPM assessment performed at the Run-in Visit started during that visit and participants returned the ABPM device after assessment completion (after completing a minimum of 25-hour ABPM readings).
- Week 12 (visit 7, end of part 1, randomized, double-blind period): the ABPM device was collected by participants at the study site the day before the visit and ABPM measurements were then started. The device was returned by the participants after completion of the ABPM assessment (after completing a minimum of 25-hour ABPM readings) during the week 12 visit.

Pharmacodynamic Analyses

Blood samples for pharmacodynamic analyses were collected at prespecified visits in the morning, after the participant had been out of bed for more than 2 hours. The participant had to be seated for 5 to 15 minutes prior to blood extraction. Hormone measurements, including plasma renin activity (PRA) and serum aldosterone were conducted using validated assays. Aldosterone levels were measured using liquid chromatography–tandem mass spectrometry with a lower limit of quantification of 0.5 ng/dl. All blood samples were analyzed in a central laboratory that was blinded to treatment allocation during the randomized phases of the trial.

Pharmacokinetic Analyses

Pre-dose blood samples were collected for measurement of plasma concentrations of baxdrostat from all participants at week 4, week 12, week 24 and week 32. For participants who were discontinued from investigational product due to hyperkalemia, a pharmacokinetic sample was collected at the end of treatment visit. Blood samples for determination of drug concentration in plasma were assayed by LabCorp, Pharmacokinetic samples were analysed only for participants on active treatment. Placebo samples were not analysed unless there was a need to confirm that correct treatment has been given to study participants.

Statistical Methods

Interim Analyses

No interim analysis for efficacy or futility was planned. However, two clinical data locks were planned. The first one occurred after participants in both cohorts completed the 12-week double-blind period in part 1 (primary outcome) and participants in Cohort 1 completed the randomized withdrawal period in part 3 (Cohort 2 did not progress into this part). A second clinical data lock will occur when all participants in both cohorts have completed the study.

Sample Size

It was estimated that 720 participants would need to be randomized 1:1:1 in each treatment group to achieve 98% power for detecting a mean (standard deviation) difference of 6 (15) mmHg for the primary end point using a two-sample t-test with a two-sided significance level of 0.025. Using these assumptions, and assuming that 50% of randomized participants were in the resistant hypertension subpopulation, a power of 80% was expected for the secondary end point in the resistant hypertension subpopulation. Assuming an attrition rate of ≤20% during the study periods, it was estimated that the study would have 86% power to detect a 6 mmHg difference in change in seated-SBP from the randomized withdrawal period baseline (week 24) to week 32 for baxdrostat 2 mg versus placebo.

Approach to Multiple Imputation

In the primary analysis, the intercurrent events of treatment discontinuation and initiation of rescue therapy was handled via a treatment policy strategy, i.e., all available measurements were included regardless of these intercurrent events. Missing data after treatment discontinuation were imputed using a retrieved dropout method based on a missing not at random (MNAR) assumption.[1] The multiple imputation retrieved dropout (MI-RD) method assumed that the pattern of end point measurement in participants who discontinued treatment and subsequently did not have an end point measurement was similar to the pattern in participants in the same treatment group who discontinued treatment but

continued in the study and had end point measurements at subsequent visits available (i.e., retrieved dropouts). Thus, for these participants the imputation of the missing end point measurement was informed by the retrieved dropouts in the same treatment group.

Missing data after initiation of rescue therapy were imputed with a reference-based imputation method, multiple imputation washout (MI-WO).[2] The imputation method assumed that the pattern of end point measurements in participants in the baxdrostat group who received rescue therapy and subsequently did not have end point measurements was similar to the pattern in participants in the placebo group (reference group). For participants in the baxdrostat group, missing end point measurements were imputed based on observed end point measurements in participants in the placebo group assuming data pattern follows MNAR. For participants in the placebo group, imputation of missing end point measurements was based on observed end point measurements in participants in the placebo group based on missing at random (MAR) assumption. The imputation was conducted separately for the baxdrostat group and placebo group.

Missing data due to other reasons unrelated to intercurrent events of treatment discontinuation or initiation of rescue therapy were imputed based on MAR assumption.

Intercurrent events of deaths were handled using the hypothetical strategy (i.e., as if the subject had not died). Consequently, missing data following death were imputed using appropriate methods based on any preceding intercurrent events (e.g., treatment discontinuation, initiation of rescue medications) or other reasons unrelated to intercurrent events of treatment discontinuation or initiation of rescue therapy.

Sensitivity Analyses of the Primary End point

Multiple Imputation Washout for Treatment Discontinuation

MI-WO, instead of MI-RD, was used to impute missing data at week 12 following treatment discontinuation; MI-WO continued to be used for missing data at week 12 following initiation of rescue medication.

Mixed Model for Repeated Measures (MMRM)

In the MMRM model, missing post-baseline SBP assessments were implicitly handled in the likelihood-based parameter estimation assuming values in participants with missing data were similar to those in participants with observed data with the same treatment assignment and covariate values (i.e., MAR). The model used change from baseline in SBP at each visit as the response variable, baseline SBP as a continuous fixed effect covariate, and hypertension at baseline (uncontrolled hypertension, resistant hypertension), treatment, visit, and treatment-by-visit interaction as fixed effect factors. The model was fitted using PROC MIXED in SAS assuming an unstructured covariance structure with the Kenward-Roger correction applied to obtain the degrees of freedom. All available SBP measurements, regardless of treatment discontinuation or initiation of rescue medication, were included in the analysis model.

Methods for Multiplicity Control

A multiple testing procedure was used to control the familywise Type I error rate at 0.05 (two-sided), between the primary and secondary end points. The dual primary end points were tested simultaneously at a two-sided significance level of 0.025. If both were rejected at their respective significance level, the first secondary end point was tested at a two-sided significance level of 0.05. If only one of the dual primary end points was rejected, the first secondary end point was tested using 0.025. Secondary end points were tested in a pre-defined sequence, and alpha was retained for the next hypothesis until a test failed to reject the null hypothesis or until all listed null hypotheses for secondary end points were rejected. In the multiple testing procedure, P values from all end points after the first nonrejected null hypothesis for a secondary end point are not adjusted and are considered explorative.

The secondary hypotheses were tested in the following order:

1) Difference in mean change from randomized withdrawal baseline (week 24) in seated-SBP at week 32 between participants receiving baxdrostat 2 mg versus participants receiving placebo.
2) Difference in mean change from baseline in seated-SBP at week 12 between participants receiving baxdrostat 2 mg versus participants receiving placebo in the resistant hypertension subpopulation.
3) Difference in mean change from baseline in seated-DBP at week 12 between participants receiving baxdrostat 2 mg versus participants receiving placebo.
4) Difference in the proportion of participants achieving seated-SBP <130 mmHg at week 12 between participants receiving baxdrostat 2 mg versus participants receiving placebo.
5) Difference in mean change from baseline in seated-SBP at week 12 between participants receiving baxdrostat 1 mg versus participants receiving placebo in the resistant hypertension subpopulation.
6) Difference in mean change from baseline in seated-DBP at week 12 between participants receiving baxdrostat 1 mg versus participants receiving placebo.
7) Difference in the proportion of participants achieving seated-SBP <130 mmHg at week 12 between participants receiving 1 mg baxdrostat versus participants receiving placebo.

For exploratory end points and subgroup analyses, no adjustment was made for multiplicity. Where results are reported as point estimates and 95% confidence intervals (95% CI), the widths of the confidence intervals have not been adjusted for multiplicity and should not be used to infer definitive treatment effects. P values are not presented for exploratory endpoints or subgroup analyses.

Analysis of Secondary, Pharmacodynamic and Pharmacokinetic End Points

The change in seated-SBP from week 24 to week 32 in part 3 was analyzed using analysis of covariance (ANCOVA) with treatment and hypertension status (uncontrolled, resistant) at baseline as factors and randomized withdrawal baseline (week 24) seated-SBP as a covariate. For change in seated-SBP from baseline to week 12 in the resistant hypertension subpopulation, data were analyzed using ANCOVA with treatment as a factor and baseline seated-SBP as a covariate. Change in seated-DBP from baseline to week 12 was analyzed similarly to the primary end point. The proportion of patients with seated-SBP <130 mmHg at week 12 was analyzed with a logistic regression model with baseline seated-SBP as a covariate and hypertension status (uncontrolled, resistant) as a factor. Pharmacodynamic and pharmacokinetic variables were summarized descriptively. A multiple testing procedure was used to control the familywise Type I error rate at 0.05 (two-sided) between the primary and secondary end points (see Methods for Multiplicity Control above).

Supplementary Results – Exploratory End Points

Ambulatory Blood Pressure

A total of 56 participants completed the ABPM analysis at 34 sites. Twenty received baxdostat 1 mg, 17 received baxdrostat 2 mg and 19 received placebo. The least-squares mean placebo-adjusted changes in 24-hour average SBP from baseline to week 12 were −14.6 mm Hg (95% CI, −23.0 to −6.3) in the baxdrostat 1 mg group and −16.9 mm Hg (95% CI, −25.6 to −8.3) in the baxdrostat 2 mg group (Fig. S4).

The least-squares mean placebo-adjusted change in night-time average SBP from baseline to week 12 was −11.7 mm Hg (95% CI, −19.5 to −3.8) in the pooled baxdrostat 1 mg and 2 mg group (Fig. S4).

Pharmacodynamic Measures

The median change in serum aldosterone concentration from baseline to week 12 was –4.2 ng/dl (interquartile range [IQR] 5.9) in those receiving baxdrostat 1 mg (n=161), –4.7 (5.8) ng/dl in those receiving baxdrostat 2 mg (n=146), and +0.2 (5.1) ng/dl in those receiving placebo (n=167). Over the same period, median changes in PRA were +2.7 ng/ml/hour (IQR 9.1), +2.2 (8.5) ng/ml/hour, and 0.0 (1.8) ng/ml/hour in those receiving baxdrostat 1 mg (n=138), 2 mg (n=122) and placebo (n=119), respectively. Median changes in aldosterone to PRA ratios were –3.3 ng/dl per ng/ml/hour (IQR 8.2), –3.4 (10.6) ng/dl per ng/ml/hour and –0.4 (4.5) ng/dl per ng/ml/hour for baxdrostat 1 mg (n=91), 2 mg (n=82) and placebo (n=89), respectively.

The median change in serum aldosterone concentration from randomized withdrawal period baseline (week 24) to week 32 was +0.1 ng/dl (IQR 1.8) in those receiving baxdrostat 2 mg (n=62) and +2.5 (5.0) ng/dl in those receiving placebo (n=41). Over the same period, median changes in PRA were –0.1 ng/ml/hour (IQR 8.5) and –1.2 (10.5) ng/ml/hour in those receiving baxdrostat 2 mg (n=82) and placebo (n=43), respectively. Median changes in

aldosterone to PRA ratios were +0.0 ng/dl per ng/ml/hour (IQR 0.5) and +0.6 (1.0) ng/dl per ng/ml/hour for baxdrostat 2 mg (n=24) and placebo (n=19), respectively.

Serum aldosterone levels and PRA over time are shown in Fig. S5 and Fig. S6, respectively.

Pharmacokinetic Analysis

Pre-dose plasma concentrations of baxdrostat remained consistent over time and proportionate to the baxdrostat dose through parts 1, 2 and 3 of the study (Table S7). Values below the lower limit of quantification (0.05 ng/ml) were recorded for ≤10% samples consistent with good adherence to study drug throughout.

Figure S1. Study Design.

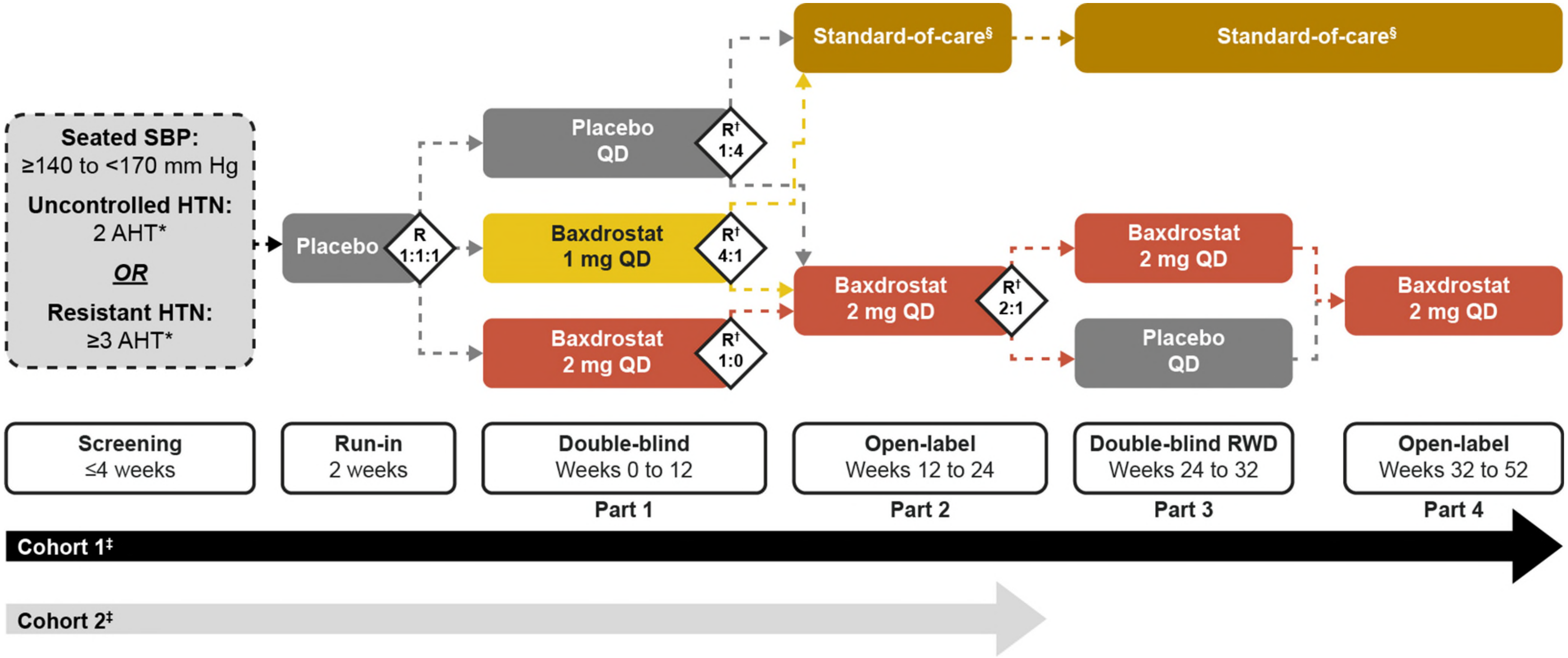

*Stable regimen of AHT from different therapeutic classes (including a diuretic), at maximum tolerated dose, for ≥4 weeks prior to screening.

†In the first open-label phase (weeks 12 to 24), participants who had received baxdrostat 2 mg in weeks 0 to 12 continued treatment; those who had received baxdrostat 1 mg or placebo were re-randomized to receive either baxdrostat 2 mg or standard-of-care (in a 4:1 or 1:4 ratio,

respectively). In the double-blind RWD phase (weeks 24 to 32), participants who had received baxdrostat 2 mg in weeks 12 to 24 were re-randomized in a 2:1 ratio to baxdrostat 2 mg or placebo.

‡Cohort 1 comprises earlier participants recruited into the trial (~n=450), who will remain in the study for 52 weeks and progress through all four parts of the study. Cohort 2 comprises participants recruited later in the trial (~n=270) and will remain in the study for 24 weeks (to end of part 2), to provide additional safety data to this timepoint. Cohort 2 participate in parts 1 and 2 of the study only, minimizing the number of participants exposed to placebo. In both cohorts, participants enter a 2-week safety follow-up at the end of the treatment phases. Full descriptions are provided in the protocol and Flack JM, Azizi M, Brown JM et al. *Hypertens Res.* 2025 DOI: 10.1038/s41440-025-02297-7.[3]

§The standard-of-care arm was included to provide reference safety data during periods with open-label baxdrostat treatment and minimize long-term exposure to placebo. Patients followed the same visit schedule as the treatment arm and continued their background medication. Mineralocorticoid receptor antagonists were allowed in the standard-of-care cohort.

AHT denotes antihypertensive treatments, HTN hypertension, QD once daily, R randomization, RWD randomized withdrawal, and SBP systolic blood pressure.

Figure S2. Participant Disposition from Screening to Week 12 (A), and Throughout the Rest of the Study (B).

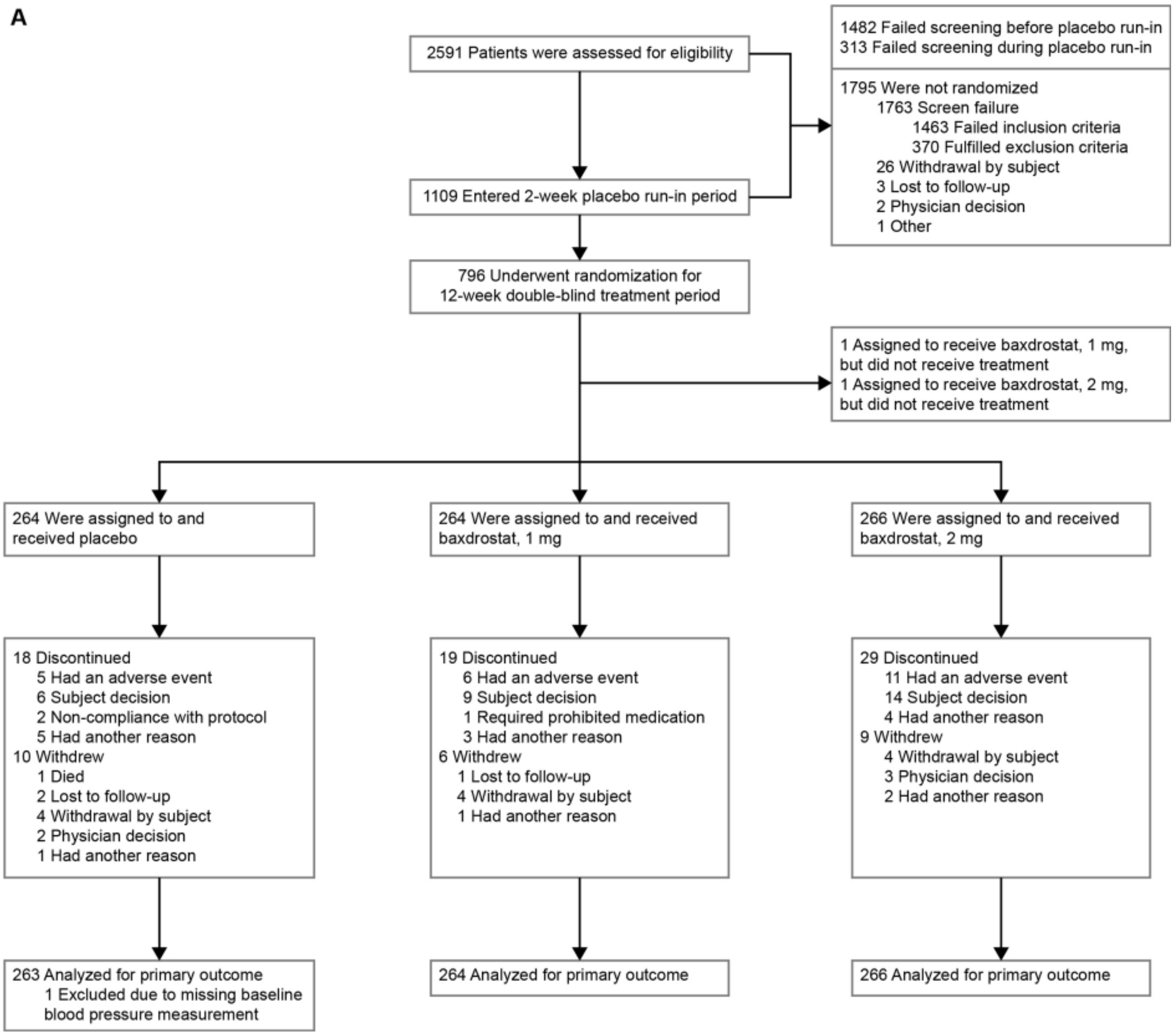

B

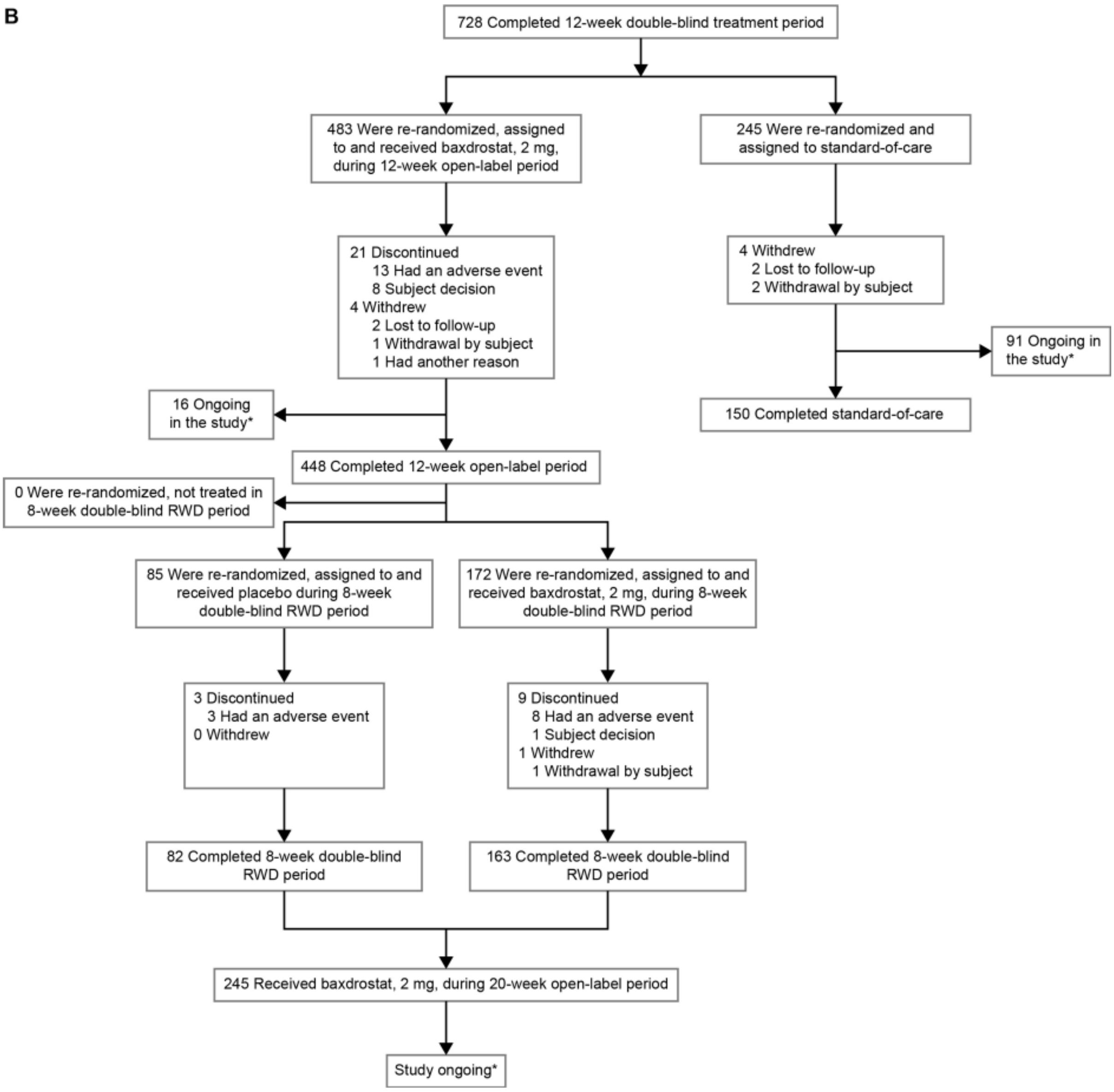

The primary efficacy analyses were assessed at the end of the 12-week double-blind period (shown in panel A). One participant in the placebo group had a missing baseline blood pressure measurement and was excluded from the primary outcome analysis. Cohort 2 completed the trial at week 24 after part 2 (first open-label period) and did not enter the double-blind RWD period.

*At the time of the primary data lock, some participants from Cohort 2 were ongoing in the first open-label phase of the study (part 2). The first double-blind treatment (part 1) and RWD (part 3) period were complete for efficacy and are reported in this manuscript.

RWD denotes randomized withdrawal.

Figure S3. Change from Baseline in Seated Systolic Blood Pressure at Week 12 by Subgroup for Baxdrostat 1 mg versus Placebo**.**

Subgroup		Baxdrostat 1 mg n	Placebo n	Least-squares mean difference (95% CI) in seated systolic blood pressure – mm Hg
**Overall**		264	263	−8.7 (−11.5, −5.8)
**Age – years**	<65	168	138	−10.3 (−14.3, −6.4)
	≥65 to <75	71	91	−7.4 (−12.5, −2.4)
	≥75	25	34	−5.5 (−12.5, 1.5)
**Sex**	Female	95	102	−9.0 (−14.1, −3.9)
	Male	169	161	−8.6 (−12.0, −5.2)
**Race**	Black or African American	23	15	−0.5 (−11.5, 10.6)
	Asian	65	72	−7.8 (−13.1, −2.4)
	White	165	166	−9.7 (−13.2, −6.1)
**Ethnicity**	Hispanic or Latino	27	38	−14.0 (−21.5, −6.6)
	Not Hispanic or Latino	231	215	−7.6 (−10.7, −4.4)
**Geographic region**	Asia Pacific, Middle East and Africa	80	80	−9.9 (−15.0, −4.8)
	Americas	72	74	−9.2 (−14.3, −4.1)
	Europe	112	109	−7.5 (−12.0, −3.0)
**Baseline hypertension**	Uncontrolled hypertension	77	71	−7.3 (-12.4, −2.3)
	Resistant hypertension	187	192	−9.1 (−12.6, −5.7)
**Baseline systolic blood pressure – mm Hg**	<145	92	92	−8.5 (−13.4, −3.5)
	≥145	172	171	−8.9 (−12.4, −5.4)
**Baseline eGFR – ml/min/1.73m²**	<60	27	29	−7.3 (−16.6, 2.1)
	≥60	237	234	−8.8 (−11.8, −5.9)
**Baseline body-mass index – kg/m²**	<30	129	128	−8.1 (−12.1, −4.1)
	≥30	135	135	−9.1 (−13.2, −5.0)

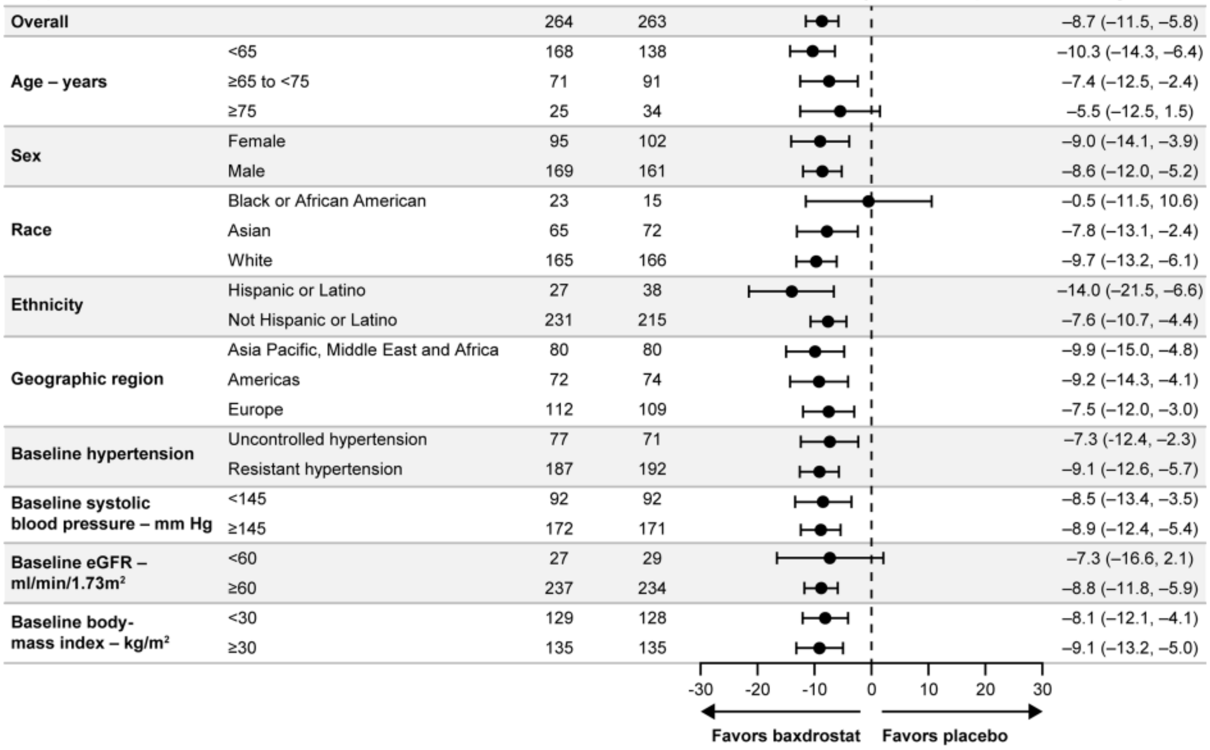

Forest plot shows least-squares mean difference versus placebo for change from baseline in seated systolic blood pressure at week 12 with baxdrostat 1 mg by subgroup. The analysis was performed within each subgroup category using an ANCOVA model with treatment and baseline hypertension (uncontrolled hypertension, resistant hypertension) as factors, and baseline seated systolic blood pressure value as a covariate. For the baseline hypertension subgroup analysis, the baseline hypertension factor was removed from the ANCOVA models for uncontrolled and resistant hypertension categories. Missing data were handled via multiple imputation. Circle denotes the point estimate. The widths of confidence intervals have not been adjusted for multiplicity and cannot be used to infer treatment effects

ANCOVA denotes analysis of covariance, CI confidence interval, and eGFR, estimated glomerular filtration rate.

Figure S4. Change from Baseline to Week 12 in Ambulatory 24-hour Average SBP mm Hg (A) and Night-time Average SBP mm Hg (B).

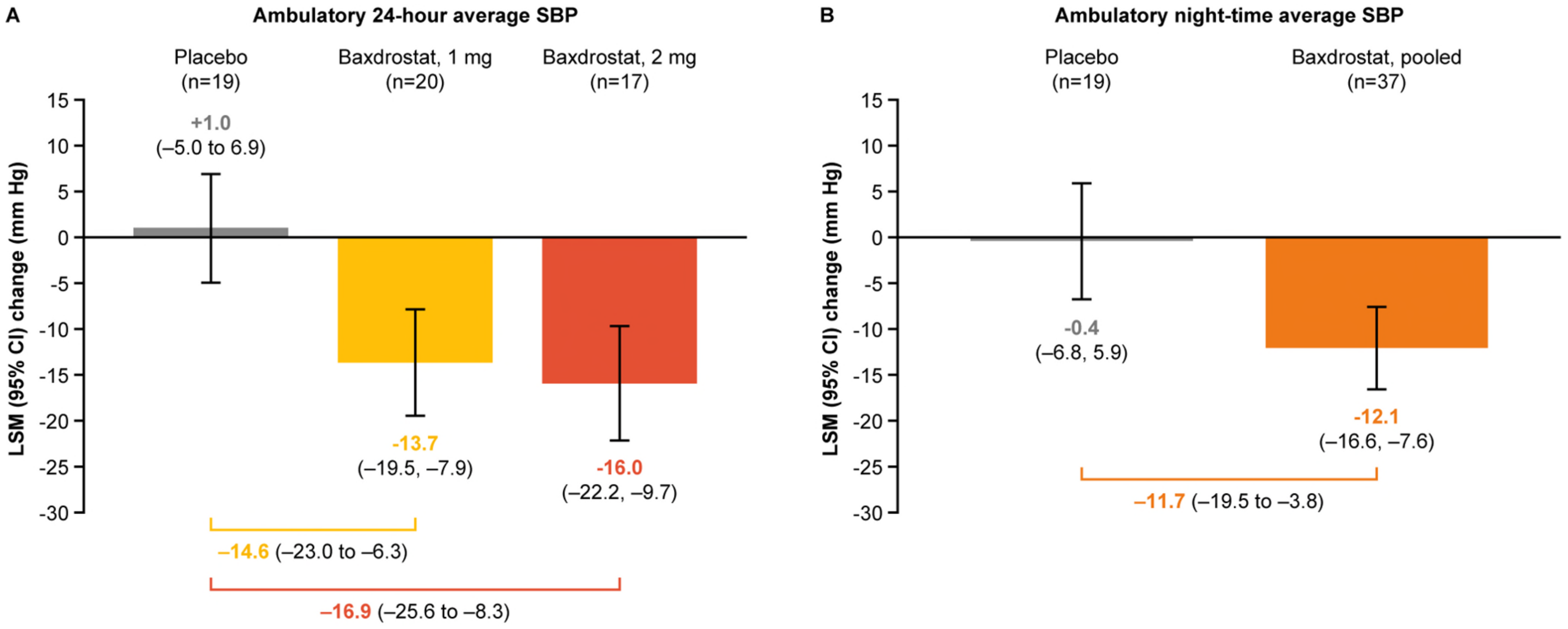

The analysis for panel A was performed using an ANCOVA model with treatment and HTN category at baseline (uHTN, rHTN) as factor, and baseline ambulatory 24-hour average SBP value as a covariate. The analysis for panel B was performed using an ANCOVA model with treatment and HTN category at baseline (uHTN, rHTN) as factor, and baseline ambulatory night-time average SBP value as a covariate.

Missing data were not imputed. I bar indicates 95% CI. The widths of confidence intervals have not been adjusted for multiplicity and cannot be used to infer treatment effects.

ANCOVA denotes analysis of covariance, CI confidence interval, HTN hypertension, LSM least-squares mean; rHTN resistant HTN, SBP systolic blood pressure and uHTN uncontrolled HTN.

Figure S5. Serum Aldosterone Levels (ng/dl) from Baseline to Week 12 (A), and from the Beginning (Week 24) to End (Week 32) of the Randomized Withdrawal Period (B).

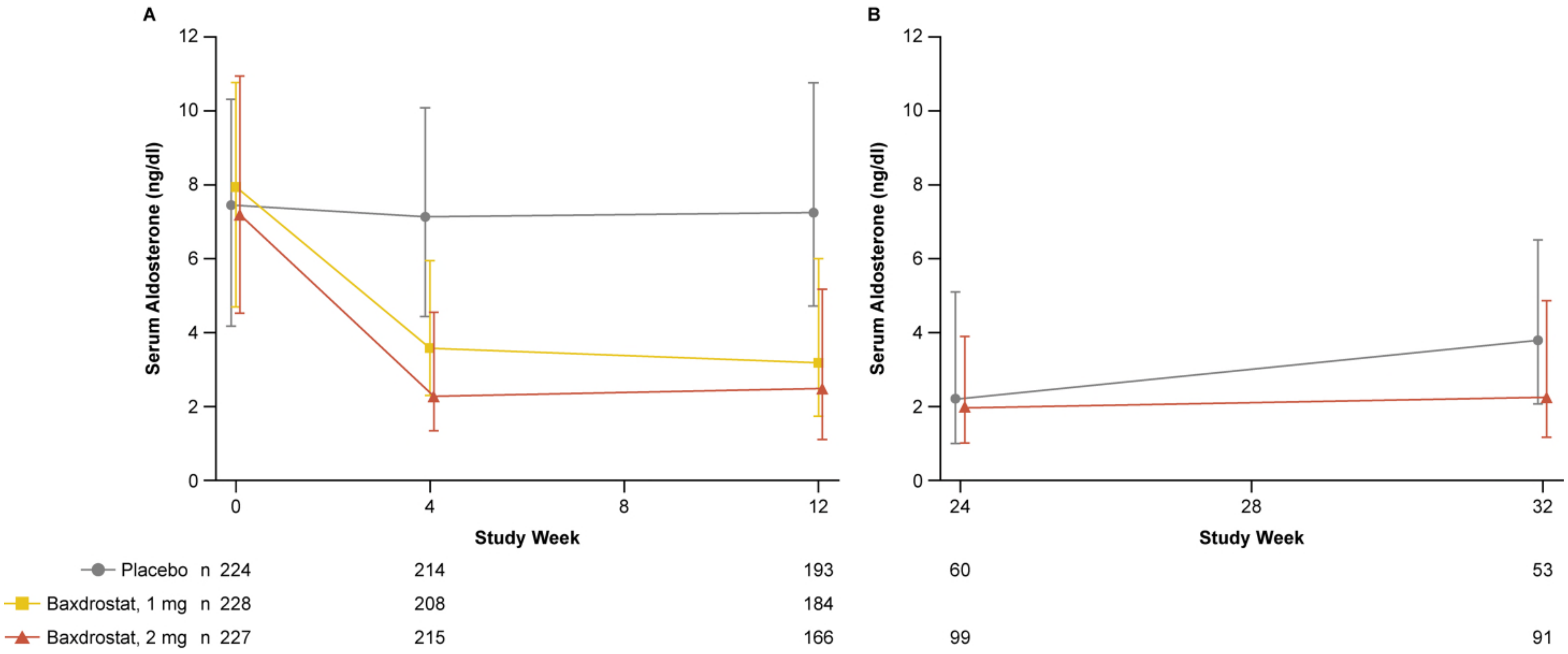

Shown is the median serum aldosterone level. I bar indicates interquartile range. n = all participants with values recorded at that timepoint.

Figure S6. Plasma Renin Activity (ng/ml/hr) from Baseline to Week 12 (A), and from the Beginning (Week 24) to End (Week 32) of the Randomized Withdrawal Period (B).

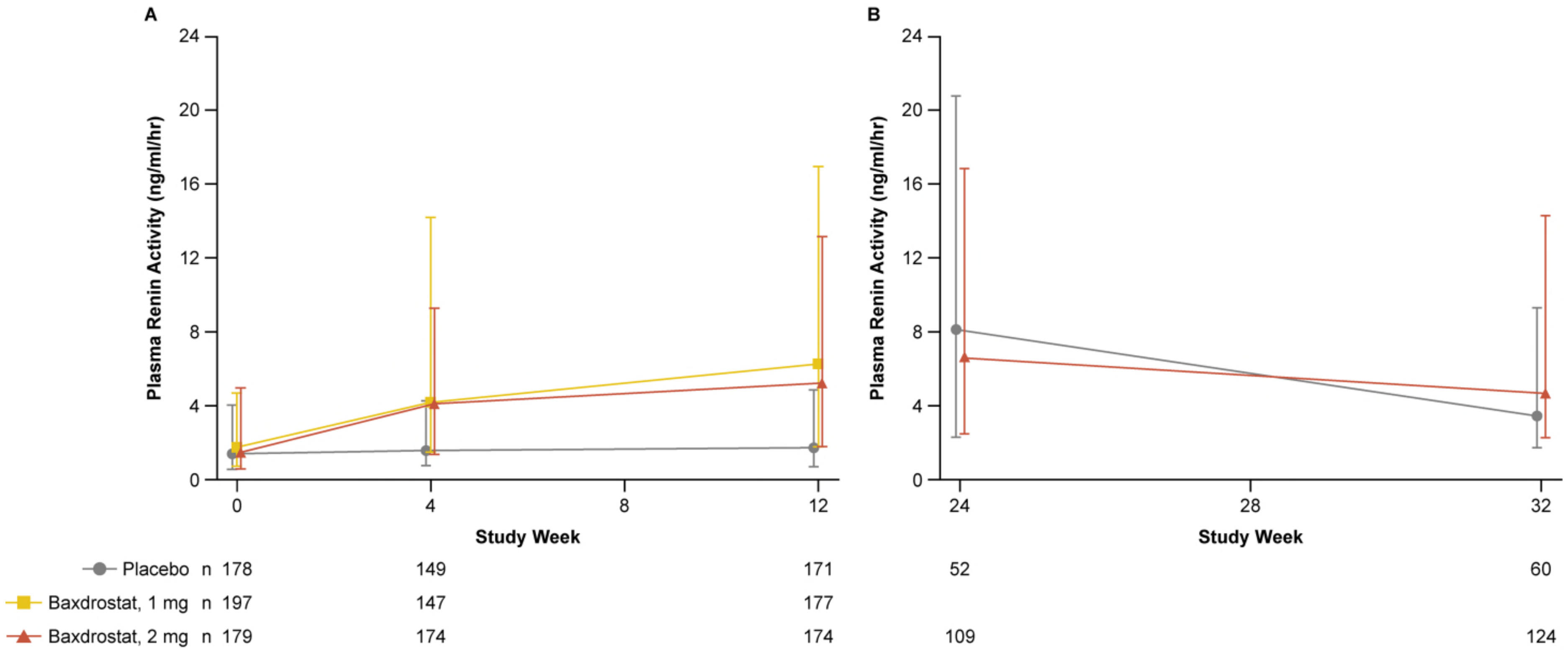

Shown is the median plasma renin activity. I bar indicates interquartile range. n = all participants with values recorded at that timepoint.

Figure S7. Serum Potassium Levels (mmol/l) from Baseline to Week 12.

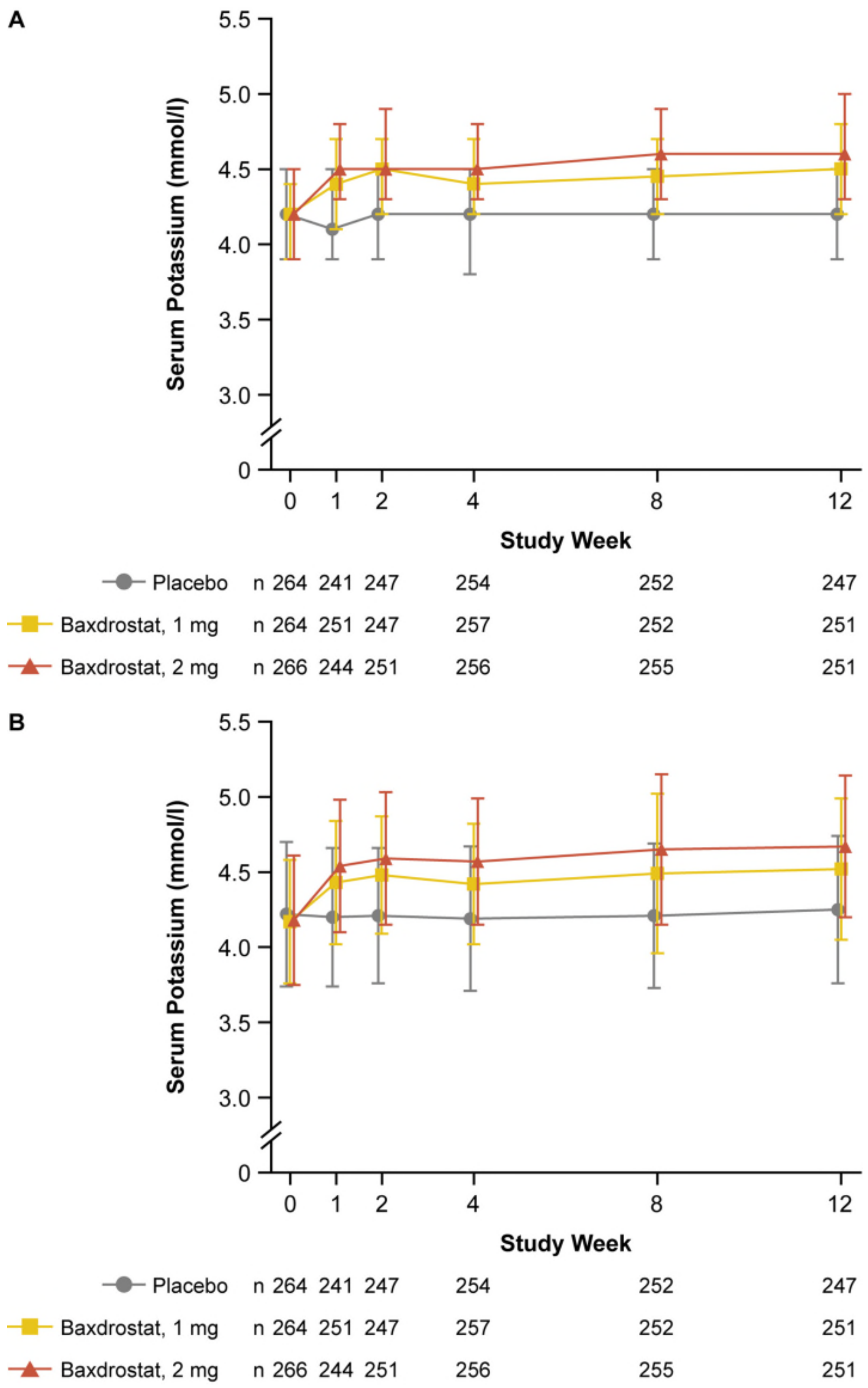

Panel A shows the median serum potassium level, with I bar indicating interquartile range.

Panel B shows the mean serum potassium level, with I bar indicating standard deviation.

n = all participants with values recorded at that timepoint.

Figure S8. Serum Sodium Levels (mmol/l) from Baseline to Week 12.

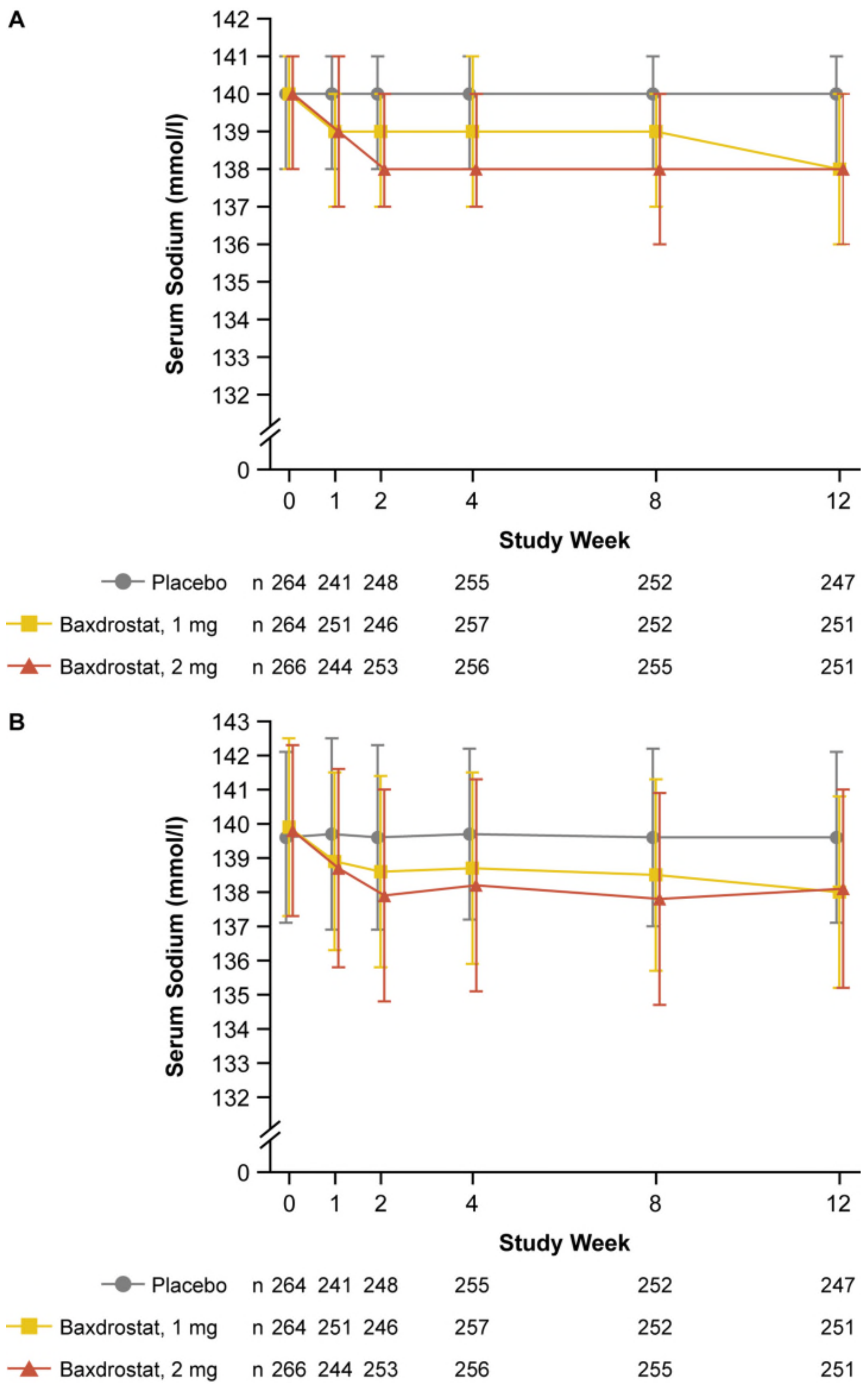

Panel A shows the median serum sodium level, with I bar indicating interquartile range.

Panel B shows the mean serum sodium level, with I bar indicating standard deviation.

n = all participants with values recorded at that timepoint.

Figure S9 Estimated Glomerular Filtration Rate from Baseline to Week 12 (A), and from the Beginning (Week 24) to End (Week 32) of the Randomized Withdrawal Period (B).

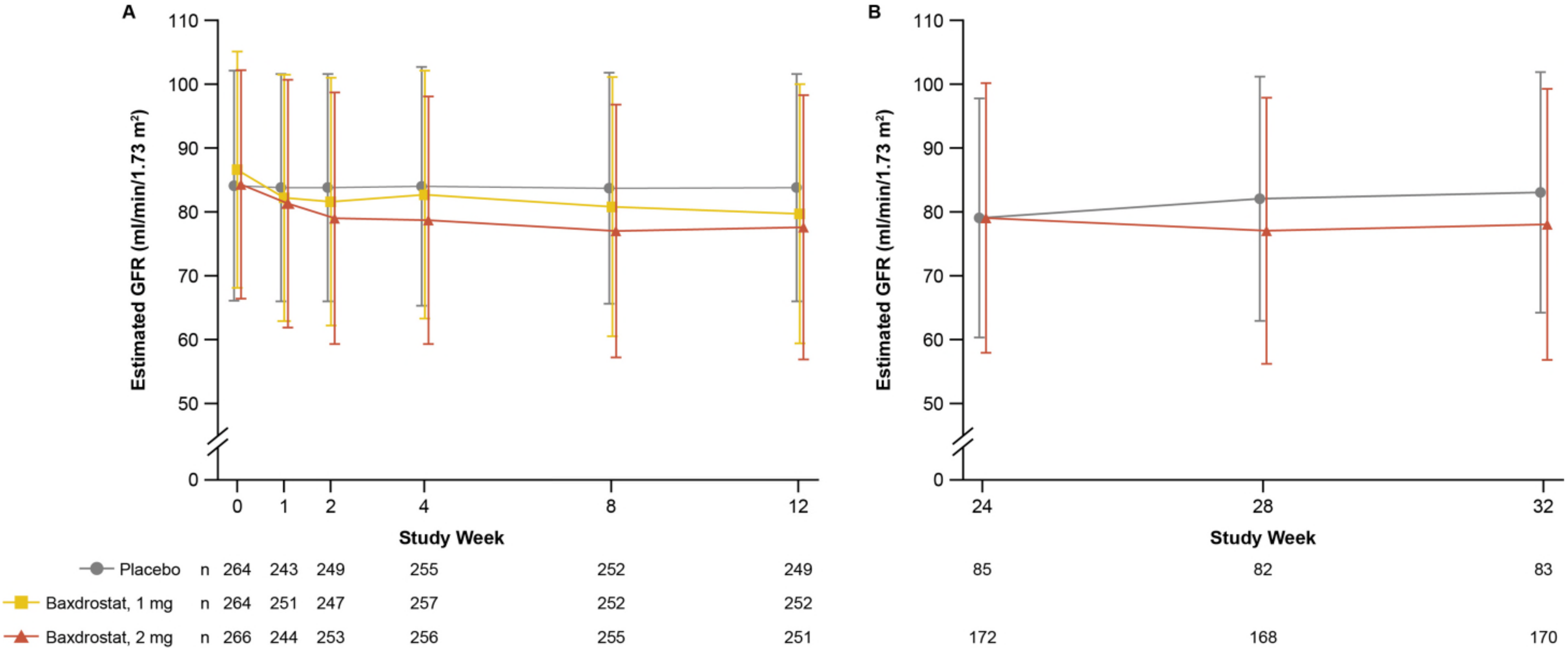

Shown is the mean estimated GFR. I bar indicates standard deviation. n = all participants with values recorded at that timepoint.

GFR denotes glomerular filtration rate.

Table S1. Full Demographics of Study Participants and their Clinical Characteristics at Baseline*

Characteristic	Placebo (N=264)	Baxdrostat, 1 mg (N=264)	Baxdrostat, 2 mg (N=266)
**Age**			
Mean – yr	61.9±11.6	59.8±11.8	61.8±11.7
<65 yr – no. (%)	139 (52.7)	168 (63.6)	145 (54.5)
≥65–<75 yr – no. (%)	91 (34.5)	71 (26.9)	88 (33.1)
≥75 yr – no. (%)	34 (12.9)	25 (9.5)	33 (12.4)
**Male sex – no. (%)**	162 (61.4)	169 (64.0)	163 (61.3)
**Race or ethnic group – no. (%)†**			
White	167 (63.3)	165 (62.5)	168 (63.2)
Black	15 (5.7)	23 (8.7)	21 (7.9)
Asian	72 (27.3)	65 (24.6)	72 (27.1)
Native Hawaiian or Pacific Islander	1 (0.4)	1 (0.4)	0 (0.0)
American Indian or Alaskan Native	0 (0.0)	0 (0.0)	0 (0.0)
Multiple	0 (0.0)	1 (0.4)	1 (0.4)
Other	7 (2.7)	8 (3.0)	1 (0.4)
Hispanic or Latino	38 (14.4)	27 (10.2)	39 (14.7)
Missing	2 (0.8)	1 (0.4)	3 (1.1)
**Geographic region – no. (%)**			

Americas	74 (28.0)	72 (27.3)	68 (25.6)
Europe	110 (41.7)	112 (42.4)	118 (44.4)
Asia Pacific, Middle East, and Africa	80 (30.3)	80 (30.3)	80 (30.1)
**Seated blood pressure – mmHg**			
n	263	264	266
Systolic	149.0±8.7	149.7±10.1	149.1±9.1
Diastolic	85.8±10.5	88.0±10.5	85.8±10.5
**Body-mass index**[‡]	31.1±6.0	31.5±6.4	31.2±6.2
Obese (body-mass index ≥30) – no. (%)	135 (51.1)	135 (51.1)	140 (52.6)
**Estimated glomerular filtration rate**[§]			
Mean – ml/min/1.73m^2	84.1±18.0	86.6±18.5	84.3±17.9
<60 ml/min/1.73m^2 – no. (%)	29 (11.0)	27 (10.2)	30 (11.3)
≥60 ml/min/1.73m^2 – no. (%)	235 (89.0)	237 (89.8)	236 (88.7)
**Diabetes – no. (%)**			
Yes	110 (41.7)	83 (31.4)	110 (41.4)
No	154 (58.3)	181 (68.6)	156 (58.6)
**Serum sodium – mmol/l**			
Mean±SD	139.6±2.5	139.9±2.6	139.8±2.5
Median (IQR)	140 (138,141)	140 (138,141)	140 (138,141)
**Serum potassium – mmol/l**			
Mean±SD	4.2±0.5	4.2±0.4	4.2±0.4

Median (IQR)	4.2 (3.9, 4.5)	4.2 (3.9, 4.4)	4.2 (3.9,4.5)
**Serum aldosterone – ng/dl**			
n	224	228	227
Mean±SD	8.2±5.1	8.6±5.7	8.5±5.5
Median (IQR)	7.5 (4.2,10.3)	7.9 (4.7, 10.8)	7.2 (4.5, 10.9)
**Plasma renin activity – ng/ml/hour**			
n	178	197	179
Mean±SD	3.7±5.9	4.4±7.2	3.9±6.3
Median (IQR)	1.4 (0.6, 4.0)	1.8 (0.7, 4.7)	1.5 (0.6, 5.0)
**Baseline hypertension¶ – no. (%)**			
Uncontrolled hypertension	71 (26.9)	77 (29.2)	67 (25.2)
Resistant hypertension	193 (73.1)	187 (70.8)	199 (74.8)
**Number of background antihypertensive medications – no. (%)**			
2	70 (26.5)	77 (29.2)	66 (24.8)
3	110 (41.7)	106 (40.2)	106 (39.8)
4	71 (26.9)	56 (21.2)	72 (27.1)
5+	13 (4.9)	25 (9.5)	22 (8.3)
**Median number of background antihypertensive medications**	3	3	3
**Background antihypertensive drug – no. (%)**			

ACE inhibitor or ARB	236 (89.4)	240 (90.9)	240 (90.2)
Beta blocker	90 (34.1)	80 (30.3)	97 (36.5)
Calcium channel blocker	177 (67.0)	184 (69.7)	191 (71.8)
Diuretic	264 (100.0)	262 (99.2)	265 (99.6)
Other	42 (15.9)	44 (16.7)	48 (18.0)

*Plus–minus values are mean ± standard deviation. Baseline characteristics are shown for the full analysis set (all randomized participants who received at least one dose of study intervention). Percentages may not total 100 because of rounding. To convert the values for sodium to milligrams, multiply by 23; to convert the values for potassium to milligrams, multiply by 39; to convert the values for aldosterone to picomoles per liter, multiply by 27.74.

†Race and ethnic group were reported by the participant. The missing numbers included in the table relate to participants whose race was not available.

‡The body-mass index is the weight in kilograms divided by the square of the height in meters.

§eGFR calculated according to CKD-EPI equation as per Inker LA et al.[4]

¶Based on information collected in the medication case report form.

ACE denotes angiotensin-converting enzyme, ARB angiotensin-receptor blocker, CKD-EPI Chronic Kidney Disease Epidemiology Collaboration, eGFR estimated glomerular filtration rate, IQR interquartile range, and SD standard deviation.

Table S2. Representativeness of the Trial Population.

Disease under investigation	**Uncontrolled and Resistant Hypertension**<br><br>Globally, hypertension (defined as SBP/DBP ≥140/90 mmHg or taking medication for hypertension) affects 33% adults aged 30–79.[5]<br><br>Definitions of *uncontrolled hypertension* (uHTN) vary. Applying the WHO definition (SBP ≥140 mmHg or DBP ≥90 mmHg), 26% of the global population have uHTN (equating to 79% individuals with HTN).[5]<br><br>*Resistant hypertension* (rHTN) is commonly defined as BP ≥140/90 mmHg in participants receiving ≥3 antihypertensive (anti-HTN) treatments and lifestyle advice: prevalence varies around 10–20% of hypertensive individuals, depending on the population and definition applied.[6]
Special considerations related to:	
Age	Overall, the prevalence of HTN increases with age.[5]<br><br>*uHTN:* in an analysis of NHANES data (2015–18) among participants with uHTN (BP ≥140/90 mmHg, aware of having HTN and treated with anti-HTN treatments), 68% of participants were ≥60 yrs of age.[7] In an Italian study using data from 2016–17, participants with uHTN (BP ≥140/90 mmHg of whom >90% were receiving ≥1 anti-HTN treatment) had a mean age of 70.[8]<br><br>*rHTN:* older age is independently associated with rHTN in most studies.[6] In the US the prevalence of rHTN (BP ≥130/80 mmHg on ≥3 classes of anti-HTN medication or on ≥4 classes regardless of BP level) was 14% among those 50–59 yrs of age, 20% among those 60–69 yrs of age, and 29% among those ≥70 yrs of age (NHANES 2009–2014).[9]<br><br>In further US and Italian studies, adults with uncontrolled BP on three anti-HTN medications (BP ≥140/90 mmHg), or on ≥4 anti-HTN medications, had a mean age of 69–70 yrs.[8,10]

Sex and gender	Globally, 34% of males and 32% of females have HTN.[5] However, proportions depend upon age: for example, the global age-standardized prevalence of HTN for those aged 30–49 yrs is 19% for women versus 24% for men, compared with 49% for both women and men aged 50–79 yrs.[5] US data suggest the percentage of women with HTN is higher than for men in participants ≥65 yrs of age.[11]<br><br>*uHTN*: 55–56% participants were female in studies among participants with BP ≥140/90 mmHg[7,8,12] and aware of having HTN[7] or with self-reported HTN and receiving anti-HTN treatment[12] or receiving ≥1 anti-HTN treatments.[8]<br><br>*rHTN:* in US and Italian studies, approximately 52–56% participants with BP ≥140/90 mmHg on ≥3 anti-HTN treatments were female.[8–10]
Race or ethnic group	In the US, the overall prevalence of HTN is highest in Black females (58%) and lowest in Hispanic females (35%) (NHANES 2017–2020, adults aged ≥20 yrs).[11] Beyond the US, data on ethnicity are limited. However, in an analysis of data from 2006 to 2010, Black (compared with White) ethnicity was associated with lack of HTN control in middle-aged adults in Great Britain.[13]<br><br>*uHTN:* among participants with BP ≥140/90 mmHg aware of having HTN and receiving anti-HTN treatment (US NHANES data 2015–18), 62% were non-Hispanic White, 18% were non-Hispanic Black, 11% were Hispanic, and 5% were non-Hispanic Asian.[7]<br><br>*rHTN:* in a further NHANES analysis (2009–2014) among participants with BP ≥130/80 mmHg on ≥3 classes of anti-HTN medication or ≥4 classes regardless of BP level, 69% were White, 21% were Black, and 7% were Hispanic.[9]
Geography	HTN is prevalent across all countries and regions of the world.[5,14] In an analysis of trends from 1990 to 2019 for 184 countries and territories, HTN

	prevalence was highest throughout central and eastern Europe, central Asia, Oceania, southern Africa, and some countries in Latin America and the Caribbean;[14] rates of uHTN follow similar patterns.[5] The greatest number of people with HTN live in the most populous WHO regions: Western Pacific Region and South-East Asia Region.[5]<br><br>Country-level data describing the characteristics of participants with uHTN and rHTN are limited. However, overall, factors associated with higher risk of rHTN (such as older age and high BMI) appear to be consistent across populations.[6]
Other considerations: presence of comorbidities	Both rHTN and uHTN are associated with the presence of comorbidities including chronic kidney disease (CKD), diabetes, obesity, and obstructive sleep apnea, which are increasing in prevalence with global population aging.[6,13]<br><br>*uHTN:* in an analysis of NHANES data (2015–18) among participants with uHTN (BP ≥140/90 mmHg, aware of having HTN and treated with anti-HTN treatment), 52% had obesity, 31% had diabetes, 42% had CKD, and 22% had a history of cardiovascular disease.[7] Similarly, European (Italian) data from 2016 to 2017 indicates 25% participants with uHTN (BP ≥140/90 mmHg receiving anti-HTN therapy) had diabetes and 4% had CKD.[8]<br><br>*rHTN:* in the same Italian study, among participants with BP ≥140/90 mmHg on ≥3 anti-HTN treatment, 34% had diabetes and 7% had CKD.[8]
Overall representativeness of this trial	Detailed epidemiological data at a regional or country level is limited and definitions of uHTN and rHTN vary. The BaxHTN trial included participants aged ≥18 yrs with either uHTN or rHTN, defined as seated-SBP between ≥140 and <170 mmHg despite two or ≥3 anti-HTN treatments, respectively.<br><br>Our population was largely consistent with observational data, where available, including mean age and presence of comorbidities. For example, approximately 40% of our population had diabetes. We recruited a slightly

	lower proportion of women and the proportion of participants with Black ethnicity was low (7%) in the total population. However, 36% of participants from North America were of Black ethnicity. Further, we recruited a balance of participants from regions across the world (27% Americas; 30% Asia, Pacific, Middle East and Africa; 43% Europe).<br><br>In BaxHTN, investigators recorded their participant's sex, age, race, and ethnicity at screening in an electronic case report form. Participant race and ethnicity were self-reported, with ethnicity asked about before race.

Anti-HTN denotes antihypertensive, BMI body mass index, BP blood pressure, CKD chronic kidney disease, DBP diastolic blood pressure, HTN hypertension, NHANES National Health and Nutrition Examination Survey, rHTN resistant HTN, SBP systolic blood pressure, uHTN uncontrolled HTN, US United States, and WHO World Health Organization.

Methodology: PubMed search to identify recent references (2022–2025) reporting the epidemiology of resistant and uncontrolled hypertension. Search terms included epidemiology, incidence, prevalence, disease burden and sex, gender and regional differences. A citation analysis was conducted to identify key sources including older references which were added manually. References were reviewed manually to identify those of relevance to the study population.

Table S3. Background Antihypertensive Treatments at Week 12.

	Placebo (N=251)	Baxdrostat, 1 mg (N=256)	Baxdrostat, 2 mg (N=251)
**Number of background antihypertensive medications – no. (%)**			
1	1 (0.4)	1 (0.4)	0 (0.0)
2	66 (26.3)	77 (30.1)	62 (24.7)
3	104 (41.4)	102 (39.8)	103 (41.0)
4	68 (27.1)	51 (19.9)	65 (25.9)
5	7 (2.8)	22 (8.6)	18 (7.2)
6	5 (2.0)	3 (1.2)	3 (1.2)
**Median number of background antihypertensive medications**	3	3	3
**Background antihypertensive drug – no. (%)***			
ACE inhibitor or ARB	223 (88.8)	232 (90.6)	225 (89.6)
Beta blocker	86 (34.3)	75 (29.3)	90 (35.9)
Calcium channel blocker	167 (66.5)	176 (68.8)	182 (72.5)
Diuretic	249 (99.2)	253 (98.8)	248 (98.8)
Mineralocorticoid receptor antagonist	4 (1.6)	0 (0.0)	0 (0.0)
Potassium-sparing diuretic	0 (0.0)	0 (0.0)	0 (0.0)

Other	40 (15.9)	43 (16.8)	46 (18.3)

*Beta blockers used for non-hypertensive indications were not counted as background antihypertensive treatment. While not permitted in part 1, mineralocorticoid receptor antagonists were permitted for patients moving to standard of care at the week 12 visit (end of part 1/start of part 2 of the study).

Data is per collected concomitant medications.

Percentages may not total 100 because of rounding.

Background antihypertensive medication classes are based on the Anatomical Therapeutic Chemical codes.

ACE denotes angiotensin-converting enzyme, and ARB angiotensin-receptor blocker.

Table S4. Missing Data for the Primary End Point.

	Placebo (N=264)	Baxdrostat, 1 mg (N=264)	Baxdrostat, 2 mg (N=266)
Full analysis set with non-missing baseline SBP, n	263	264	266
Participants with missing Week 12 SBP – no. (%)	18 (6.8)	13 (4.9)	16 (6.0)
Following treatment discontinuation – no. (%)	5 (1.9)	8 (3.0)	4 (1.5)
Following initiation of rescue medication – no. (%)	0 (0.0)	0 (0.0)	1 (0.4)
Unrelated to treatment discontinuation or initiation of rescue medication – no. (%)	13 (4.9)	5 (1.9)	11 (4.1)

SBP denotes systolic blood pressure.

Table S5. Blood Pressure Changes with Baxdrostat – Full Results for Primary Outcome and Secondary Outcomes (According to Hierarchical Order).

End Point	Placebo	Baxdrostat, 1 mg	Baxdrostat, 2 mg
**Primary end point – change in seated-SBP from baseline to week 12***			
n	263	264	266
LS mean (95% CI) – mmHg	–5.8 (–7.9 to –3.8)	–14.5 (–16.5 to –12.5)	–15.7 (–17.6 to –13.7)
LS mean placebo-corrected difference (95% CI) – mmHg	–	–8.7 (–11.5 to –5.8)	–9.8 (–12.6 to –7.0)
P value	–	<0.0001	<0.0001
**Secondary end point – change in seated-SBP from randomized withdrawal period baseline (week 24) to week 32 (baxdrostat 2 mg versus placebo)†**			
n	85	NA	172

LS mean (95% CI) – mmHg	1.4<br>(–1.2 to 4.0)	NA	–3.7<br>(–5.5 to –1.9)
LS mean placebo-corrected difference (95% CI) – mmHg	–	NA	–5.1<br>(–8.3 to –1.9)
P value	–	NA	0.0016
**Secondary end point – change in seated-SBP from baseline to week 12 in the resistant hypertension subpopulation‡**			
n	192	187	199
LS mean (95% CI) – mmHg	–5.4<br>(–7.9 to –2.9)	–14.5<br>(–17.0 to –12.1)	–15.2<br>(–17.4 to –12.9)
LS mean placebo-corrected difference (95% CI) – mmHg	–	–9.1<br>(–12.6 to –5.7)	–9.8<br>(–13.1 to –6.4)
P value	–	<0.0001	<0.0001
**Secondary end point – change in seated-DBP from baseline to week 12§**			
n	263	264	266

LS mean (95% CI) – mmHg	–3.0<br>(–4.4 to –1.7)	–6.3<br>(–7.7 to –4.9)	–6.9<br>(–8.1 to –5.6)
LS mean placebo-corrected difference (95% CI) – mmHg	–	–3.3<br>(–5.2 to –1.4)	–3.9<br>(–5.7 to –2.0)
P value	–	0.0008	<0.0001
**Secondary end point – achieving seated-SBP <130 mmHg at Week 12¶**			
n	262	264	265
n (%) achieving seated-SBP <130 mmHg	49 (18.7)	104 (39.4)	106 (40.0)
Odds ratio (95% CI)	–	2.9 (1.9, 4.3)	2.9 (1.9, 4.4)
P value	–	<0.0001	<0.0001

*The analysis was performed on the full analysis set using an ANCOVA model with treatment and hypertension at baseline (uncontrolled hypertension, resistant hypertension) as factors, and baseline seated-SBP value as a covariate.

†The analysis was performed on the randomized withdrawal set as a pre-specified analysis for baxdrostat 2 mg versus placebo only, using an ANCOVA model with treatment and hypertension at baseline (uncontrolled hypertension, resistant hypertension) as factors, and randomized withdrawal period baseline seated-SBP value as a covariate.

‡The analysis was performed on the full analysis set using an ANCOVA model with treatment as factor, and baseline seated-SBP value as a covariate. Only participants with resistant hypertension at baseline were included in the analysis.

§The analysis was performed on the full analysis set using an ANCOVA model with treatment and hypertension at baseline (uncontrolled hypertension, resistant hypertension) as factors, and baseline seated-DBP value as a covariate.

¶The analysis was performed using a logistic regression model with baseline seated-SBP value as a covariate and treatment and hypertension at baseline (uncontrolled hypertension, resistant hypertension) as factors. The analysis includes participants with baseline seated-SBP of 130 mmHg or greater. An odds ratio greater than 1 favors baxdrostat.

For all analyses except change in seated-SBP from randomized withdrawal period baseline (week 24) to week 32, missing data at week 12 following treatment discontinuation were imputed using a multiple imputation retrieved dropout method and missing data at week 12 following initiation of rescue medication were imputed using a multiple imputation washout method. For change in seated-SBP from randomized withdrawal period baseline (week 24) to week 32, a multiple imputation washout method was used at week 32 for missing data following both treatment discontinuation and rescue medication. Intercurrent events of deaths were handled using the hypothetical strategy (i.e., as if the subject had not died).

P value is from the ANCOVA model for all analyses, except achieving seated-SBP <130 mmHg at week 12, where the p value is from the logistic regression model.

ANCOVA denotes analysis of covariance, CI confidence interval, DBP diastolic blood pressure, LS least-squares, NA not applicable and SBP systolic blood pressure.

Table S6. Sensitivity Analyses for the Primary End Point (Full Analysis Set).

	Placebo (N=264)	Baxdrostat, 1 mg (N=264)	Baxdrostat, 2 mg (N=266)
**Sensitivity analysis 1 – multiple imputation washout method for treatment discontinuation***			
n	263	264	266
LS mean (95% CI) – mmHg	–6.2 (–8.1 to –4.3)	–14.2 (–16.1 to –12.3)	–15.6 (–17.4 to –13.7)
LS mean placebo-corrected difference (95% CI) – mmHg		–8.0 (–10.6 to –5.3)	–9.4 (–12.0 to –6.7)
**Sensitivity analysis 2 – MMRM†**			
n	245	251	250
LS mean (95% CI) – mmHg	–6.2 (–8.1 to –4.3)	–14.6 (–16.5 to –12.7)	–15.6 (–17.5 to –13.8)
LS mean placebo-corrected difference (95% CI) – mmHg		–8.4 (–11.1 to –5.8)	–9.5 (–12.1 to –6.8)

*Analysis performed using an ANCOVA model with treatment and hypertension at baseline (uncontrolled hypertension, resistant hypertension) as factors, and baseline seated-SBP value as a covariate. Missing data at week 12 following treatment discontinuation were imputed using multiple imputation washout method and missing data at week 12 following initiation of rescue medication were imputed using multiple imputation washout method. The

widths of confidence intervals have not been adjusted for multiplicity and cannot be used to infer treatment effects.

†Analysis performed using a MMRM model with change from baseline in seated-SBP at each visit as the response variable, baseline seated-SBP as a continuous fixed effect covariate, and hypertension at baseline (uncontrolled hypertension, resistant hypertension), treatment, visit, and treatment-by-visit interaction as fixed effect factors. All available seated-SBP measurements, regardless of treatment discontinuation or initiation of rescue medication, were included in the analysis model. The widths of confidence intervals have not been adjusted for multiplicity and cannot be used to infer treatment effects.

ANCOVA denotes analysis of covariance, CI confidence interval, LS least-squares, MMRM mixed model for repeated measures, and SBP systolic blood pressure.

Table S7. Plasma Concentration of Baxdrostat Over Time (ng/ml).

Group timepoint (Pre-dose)	n<LLOQ (%)	Arithmetic mean±SD	Geometric mean±SD	Geometric CV%	Min	Median	Max
**Baxdrostat 1 mg**							
Week 4 (n=234)	15 (6.4)	12.839±6.788	10.794±2.034	80.99	0.07	11.732	35.74
Week 12 (n=224)	23 (10.3)	12.929±7.238	10.924±1.998	78.42	0.12	11.824	64.62
**Baxdrostat 2 mg**							
Week 4 (n=238)	13 (5.5)	24.321±11.190	20.320±2.248	96.33	0.05	23.018	57.15
Week 12 (n=224)	16 (7.1)	25.104±12.095	20.789±2.286	99.03	0.05	24.104	73.90
Week 24 (n=281)	20 (7.1)	26.425±12.457	22.994±1.902	71.51	0.08	24.873	72.84
Week 32 (n=122)	12 (9.8)	24.921±12.240	21.392±1.906	71.85	0.57	22.787	64.55

For each visit, all participants who were taking the relevant dose at the visit are included.

Geometric mean is the exponentiation of the arithmetic mean of the natural log-transformed values. Geometric SD is the exponentiation of the standard deviation of the natural log-transformed values. Geometric CV% is calculated as the square root of (exp(s^2)-1)*100, where s is the standard deviation of the natural log-transformed data.

CV% denotes coefficient of variation, LLOQ lower limit of quantification (0.05 ng/ml), and SD standard deviation.

Table S8. Serious Adverse Events by System Organ Class and Preferred Term During the 12-Week Double-Blind Treatment Period.

Event	Placebo (N=264)	Baxdrostat, 1 mg (N=264)	Baxdrostat, 2 mg (N=266)
		Number (percent)	
Any serious adverse event	7 (2.7)	5 (1.9)	9 (3.4)
System organ class<br>MedDRA preferred term*			
Infections and infestations	2 (0.8)	0 (0.0)	3 (1.1)
Lower respiratory tract infection	0 (0.0)	0 (0.0)	1 (0.4)
Septic shock	2 (0.8)	0 (0.0)	0 (0.0)
Typhoid fever	0 (0.0)	0 (0.0)	1 (0.4)
Urinary tract infection	0 (0.0)	0 (0.0)	1 (0.4)
Neoplasms benign, malignant and unspecified	0 (0.0)	0 (0.0)	1 (0.4)
Adenocarcinoma of colon	0 (0.0)	0 (0.0)	1 (0.4)
Metabolic and nutrition disorders	2 (0.8)	2 (0.8)	1 (0.4)
Hyperkalemia	0 (0.0)	1 (0.4)	0 (0.0)
Hypervolemia	2 (0.8)	0 (0.0)	0 (0.0)
Hyponatremia	0 (0.0)	1 (0.4)	1 (0.4)
Nervous system disorders	0 (0.0)	2 (0.8)	0 (0.0)

Altered state of consciousness	0 (0.0)	1 (0.4)	0 (0.0)
Sciatica	0 (0.0)	1 (0.4)	0 (0.0)
Cardiac disorders	2 (0.8)	0 (0.0)	0 (0.0)
Myocardial infarction	1 (0.4)	0 (0.0)	0 (0.0)
Sinus node dysfunction	1 (0.4)	0 (0.0)	0 (0.0)
Vascular disorders	0 (0.0)	0 (0.0)	2 (0.8)
Hypertension	0 (0.0)	0 (0.0)	2 (0.8)
Respiratory, thoracic and mediastinal disorders	1 (0.4) †	0 (0.0)	0 (0.0)
Acute respiratory distress syndrome	1 (0.4)	0 (0.0)	0 (0.0)
Acute respiratory failure	1 (0.4)	0 (0.0)	0 (0.0)
Pulmonary hypertension	1 (0.4)	0 (0.0)	0 (0.0)
Gastrointestinal disorders	3 (1.1) †	0 (0.0)	3 (1.1)
Diarrhea	0 (0.0)	0 (0.0)	1 (0.4)
Dyspepsia	0 (0.0)	0 (0.0)	1 (0.4)
Gastritis	1 (0.4)	0 (0.0)	0 (0.0)
Hemorrhoids	1 (0.4)	0 (0.0)	0 (0.0)
Inguinal hernia	1 (0.4)	0 (0.0)	0 (0.0)
Obstructive pancreatitis	1 (0.4)	0 (0.0)	0 (0.0)
Small intestinal obstruction	0 (0.0)	0 (0.0)	1 (0.4)
General disorders and administration site conditions	0 (0.0)	1 (0.4)	0 (0.0)

Non-cardiac chest pain	0 (0.0)	1 (0.4)	0 (0.0)
Injury, poisoning and procedural complications	1 (0.4)	0 (0.0)	0 (0.0)
Lumbar vertebral fracture	1 (0.4)	0 (0.0)	0 (0.0)

*Adverse events were coded using the preferred term in the Medical Dictionary for Regulatory Activities (MedDRA), version 28. The table includes adverse events with an onset date on or after randomisation in the initial double-blind period. Subjects were censored at the latest of the last visit within period or the disposition date within period.

†One participant in these categories reported severe adverse events under more than one preferred term.

Table S9. Adverse Events by System Organ Class and Preferred Term (Reported in >2% Subjects in Any Treatment Group) During the 12-Week Double-Blind Treatment Period.

Event	Placebo (N=264)	Baxdrostat, 1 mg (N=264)	Baxdrostat, 2 mg (N=266)
		Number (percent)	
Any adverse event	109 (41.3)	125 (47.3)	119 (44.7)
System organ class			
MedDRA preferred term*			
Infections and Infestations	41 (15.5)	36 (13.6)	28 (10.5)
Nasopharyngitis	6 (2.3)	8 (3.0)	8 (3.0)
Upper respiratory tract infection	10 (3.8)	7 (2.7)	1 (0.4)
Urinary tract infection	6 (2.3)	2 (0.8)	7 (2.6)
Metabolism and nutrition disorders	15 (5.7)	24 (9.1)	45 (16.9)
Hyperkalemia	7 (2.7)	17 (6.4)	31 (11.7)
Hyponatremia	2 (0.8)	4 (1.5)	11 (4.1)
Psychiatric disorders	2 (0.8)	5 (1.9)	7 (2.6)
Nervous system disorders	14 (5.3)	23 (8.7)	20 (7.5)
Dizziness	4 (1.5)	7 (2.7)	9 (3.4)
Headache	8 (3.0)	9 (3.4)	6 (2.3)
Cardiac disorders	6 (2.3)	7 (2.7)	5 (1.9)
Vascular disorders	12 (4.5)	12 (4.5)	16 (6.0)

Hypertension	9 (3.4)	5 (1.9)	5 (1.9)
Hypotension	2 (0.8)	6 (2.3)	10 (3.8)
Respiratory, thoracic and mediastinal disorders	8 (3.0)	9 (3.4)	8 (3.0)
Gastrointestinal disorders	19 (7.2)	16 (6.1)	18 (6.8)
Diarrhea	5 (1.9)	4 (1.5)	6 (2.3)
Skin and subcutaneous tissue disorders	3 (1.1)	6 (2.3)	4 (1.5)
Musculoskeletal and connective tissue disorders	11 (4.2)	16 (6.1)	25 (9.4)
Arthralgia	2 (0.8)	1 (0.4)	6 (2.3)
Muscle spasms	1 (0.4)	6 (2.3)	10 (3.8)
Renal and urinary disorders	4 (1.5)	9 (3.4)	7 (2.6)
General disorders and administration site conditions	12 (4.5)	11 (4.2)	9 (3.4)
Investigations†	8 (3.0)	5 (1.9)	7 (2.6)
Injury, poisoning and procedural complications	6 (2.3)	8 (3.0)	2 (0.8)

*Adverse events were coded using the preferred term in the Medical Dictionary for Regulatory Activities (MedDRA), version 28. The table includes adverse events with an onset date on or after randomisation in the initial double-blind period. Subjects were censored at the latest of the last visit within period or the disposition date within period.

†Investigations include those conducted for changes in blood chemistry.

Table S10. Clinical Chemistry Treatment Emergent Abnormalities by Predefined Criteria During the 12-Week Double-Blind Treatment Period.

	Placebo (N=264)	Baxdrostat, 1 mg (N=264)	Baxdrostat, 2 mg (N=266)
		Number (percent)	
Serum potassium* – mmol/l			
<3.5	15/250 (6.0)	7/251 (2.8)	3/249 (1.2)
>5.5 mmol/l	1/260 (0.4)	16/262 (6.1)	29/261 (11.1)
>6.0 mmol/l	1/262 (0.4)	6/262 (2.3)	8/263 (3.0)
>6.5 mmol/l	1/263 (0.4)	5/262 (1.9)	1/263 (0.4)
Serum sodium* – mmol/l			
<125	1/263 (0.4)	1/262 (0.4)	4/263 (1.5)
<130	2/263 (0.8)	3/262 (1.1)	10/263 (3.8)
<135	18/256 (7.0)	49/256 (19.1)	59/259 (22.8)
Estimated glomerular filtration rate† – % decrease from baseline			
≥30%	4/263 (1.5)	33/262 (12.6)	41/263 (15.6)
≥50%	1/263 (0.4)	4/262 (1.5)	3/263 (1.1)

*Denominators are number of participants per treatment group who, at baseline, did not already fulfil the specific row-criteria; for participants with non-missing postbaseline value(s)

but missing baseline value, the baseline value is assumed to not fulfil the specific row-criteria.

†Denominators are number of participants per treatment group who had non-missing baseline value and any non-missing postbaseline value.

Table S11. Assessment of Central Laboratory Potassium Measurements >6.0 mmol/l During the 12-Week Double-Blind Treatment Period.

	**Placebo (N=264)**	**Baxdrostat, 1 mg (N=264)**	**Baxdrostat, 2 mg (N=266)**
		Number (percent)	
**Central laboratory potassium >6.0 mmol/l***			
Participants with potassium >6.0 mmol/l – no. (%)	1/262 (0.4)	6/262 (2.3)	8/263 (3.0)
Confirmed† – no. (%)	0/262 (0.0)	3/262 (1.1)	3/263 (1.1)
Not confirmed† – no. (%)	1/262 (0.4)	2/262 (0.8)	4/263 (1.5)
Undetermined† – no. (%)	0/262 (0.0)	1/262 (0.4)	1/263 (0.4)

*A prespecified algorithm was implemented to guide the management of study medication in the event of hyperkalemia, including criteria for continuation, temporary interruption, or permanent discontinuation based on serum potassium levels (5.5 to <6.0 mmol/l or ≥6.0 mmol/l). See Supplementary Methods for further information.

†For central laboratory potassium values of >6.0 mmol/l, the confirmation status was assessed based on a local laboratory potassium measurement from the same day. If both central and local laboratory potassium values were >6.0 mmol/l on the same day, the central potassium value was classified ‘confirmed’; if the local potassium value was ≤6.0 mmol/l, the central potassium value was classified ‘not confirmed’; if the local potassium value was not available, the central potassium value was classified as ‘undetermined’. For calculation purposes, only the first event per participant was included in the analysis.

Supplementary References

1. Wang S, Hu H. Impute the missing data using retrieved dropouts. BMC Med Res Methodol 2022;22:82.
2. Wang Y, Tu W, Kim Y, et al. Statistical methods for handling missing data to align with treatment policy strategy. Pharm Stat 2023;22:650–70.
3. Flack JM, Azizi M, Brown JM, et al. Baxdrostat for uncontrolled and resistant hypertension: rationale and design of the Phase 3 clinical trials BaxHTN, BaxAsia, and Bax24. Hypertens Res 2025; DOI: 10.1038/s41440-025-02297-7.
4. Inker LA, Eneanya ND, Coresh J, et al. New creatinine- and cystatin C–based equations to estimate GFR without race. New Engl J Med 2021;385:1737–49.
5. Global report on hypertension: the race against a silent killer. Geneva: World Health Organization; 2023.
6. Brant LCC, Passaglia LG, Pinto-Filho MM, et al. The burden of resistant hypertension across the world. Curr Hypertens Rep 2022;24:55–66.
7. Sakhuja S, Colvin CL, Akinyelure OP, et al. Reasons for uncontrolled blood pressure among US Adults: data from the US National Health and Nutrition Examination Survey. Hypertension 2021;78:1567–76
8. Romano S, Rigon G, Albrigi M, et al. Hypertension, uncontrolled hypertension and resistant hypertension: prevalence, comorbidities and prescribed medications in 228,406 adults resident in urban areas. A population-based observational study. Intern Emerg Med 2023;18:1951–59.
9. Carey RM. Prevalence of apparent treatment resistant hypertension in the United States: comparison of the 2008 and 2018 American Heart Association Scientific Statements on resistant hypertension. Hypertension 2019;73:424–31.
10. Sim JJ, Bhandari SK, Shi J, et al. Characteristics of resistant hypertension in a large ethnically diverse hypertension population of an integrated health system. Mayo Clin Proc 2013;88:1099–107.

11. Martin SS, Aday AW, Allen NB, et al. 2025 Heart disease and stroke statistics: a report of US and global data from the American Heart Association. Circulation 2025;151:e41–e660.
12. Chow CK, Teo KK, Rangarajan S, et al. Prevalence, awareness, treatment, and control of hypertension in rural and urban communities in high-, middle-, and low-income countries. JAMA 2013;310:959–68.
13. Tapela N, Collister J, Clifton L, et al. Prevalence and determinants of hypertension control among almost 100 000 treated adults in the UK. Open Heart 2021;8:e001461.
14. NCD Risk Factor Collaboration. Worldwide trends in hypertension prevalence and progress in treatment and control from 1990 to 2019: a pooled analysis of 1201 population-representative studies with 104 million participants. Lancet. 2021;398: 957–80.
